# Hebbian instruction of axonal connectivity by endogenous correlated spontaneous activity

**DOI:** 10.1126/science.adh7814

**Published:** 2024-08-16

**Authors:** Naoyuki Matsumoto, Daniel Barson, Liang Liang, Michael C. Crair

**Affiliations:** 1 Department of Neuroscience, Yale University School of Medicine, New Haven, CT 06510, USA.; 2 Kavli Institute for Neuroscience, Yale University, New Haven, CT 06510, USA.; 3 Wu Tsai Institute, Yale University, New Haven, CT 06510, USA.; 4 Department of Ophthalmology & Visual Science, Yale University School of Medicine, New Haven, CT 06510, USA.

## Abstract

Spontaneous activity refines neural connectivity prior to the onset of sensory experience, but it remains unclear how such activity instructs axonal connectivity with subcellular precision. We simultaneously measured spontaneous retinal waves and the activity of individual retinocollicular axons and tracked morphological changes in axonal arbors across hours in vivo in neonatal mice. We demonstrate that the correlation of an axon branch’s activity with neighboring axons or postsynaptic neurons predicts whether the branch will be added, stabilized, or eliminated. Desynchronizing individual axons from their local networks, changing the pattern of correlated activity, or blocking *N*-methyl-d-aspartate receptors all significantly altered single-axon morphology. These observations provide the first direct evidence in vivo that endogenous patterns of correlated neuronal activity instruct fine-scale refinement of axonal processes.

Neuronal activity is essential for the construction and refinement of neuronal connectivity in the mammalian central nervous system (1, 2). Activity can act passively to enable developmental cues to guide appropriate neural connections (3, 4) or instructively to directly mold the precise patterning of neural circuits. Instructive patterning by activity can occur through competition among neighboring neurons or through activity synchronization among afferents or between afferents and postsynaptic neurons (1). The refinement of neural circuits is significantly influenced by the spatiotemporal pattern of neuronal activity driven by sensory experience. For example, artificially synchronizing retinal activity between the two eyes through strobe rearing disrupts the development of the binocular map in the cortex (5), and disrupting coordinated activity in the two eyes through strabismus causes neurons to respond more monocularly and also alters the pattern of ocular dominance columns in the cortex (6). Patterned neural activity is also produced through spontaneously generated activity. Prior to the onset of sensory experience, developing sensory organs spontaneously generate neuronal activity that propagates throughout the central nervous system and synchronizes the firing of neurons within and across brain areas (1, 7–9). The specific spatiotemporal structure of this correlated spontaneous activity is crucial for the acquisition of normal receptive field properties in sensory regions of the brain and establishes the initial configuration of functional neural circuits necessary for early behavior upon onset of sensory transduction (1, 8–12).

A long-standing question is how endogenous patterns of correlated spontaneous activity instruct the precise patterning of circuit connectivity at the single-cell level. It has been postulated that circuit refinement driven by synchronous activity follows Hebbian plasticity learning rules, summarized as “cells that fire together, wire together” and “out of sync, lose your link” (13, 14). Indeed, artificially inducing synchronous and asynchronous activity by using optogenetic, stroboscopic, or paired pre- and postsynaptic stimulation drives circuit refinement in a manner consistent with Hebbian plasticity rules (5, 15–18). However, methodological limitations have impeded direct demonstration of endogenously generated patterns of spontaneous activity driving circuit refinement through Hebbian plasticity in vivo.

Circuit refinement guided by spontaneous activity has been extensively studied in retinofugal pathways. In the mammalian visual system, retinal ganglion cells (RGCs) are synchronized by waves of activity that propagate across the developing retina prior to eye opening (19–21). Retinal waves are necessary for proper retinofugal axon positioning in target areas and onto target neurons, including the development of eye-specific segregation, retinotopy, and ON/OFF receptive fields (2, 22–27). Individual RGC axons initially overshoot their targets, and then axon arbors become progressively denser and spatially restricted during the period of spontaneous retinal waves (28, 29). Blockade of retinal waves prevents the refinement of RGC axon arbors, resulting in sparse and expanded arbors (28, 30). Although these studies show the necessity of retinal waves for establishing appropriate connections in retinofugal pathways, the importance of spatiotemporal patterns of retinal waves remains elusive. Roles for patterned activity in axon refinement have been studied by in vivo real-time imaging in optically transparent zebrafish and albino *Xenopus laevis* tadpoles (17, 31–34). However, axon refinement in these nonmammalian species occurs after the onset of vision and is driven by visual experience (35). Conversely, the role and mechanism of endogenous spontaneous activity, such as retinal waves, in instructing the precise addition and pruning of individual RGC axon arbor branches in mammals remains poorly understood. In this work, by establishing a method to visualize the relationship between retinal waves and individual RGC axon branch dynamics in awake, behaving neonatal mice, we demonstrate that endogenously generated patterns of retinal waves instruct fine-scale refinement of individual RGC axons in accordance with Hebbian plasticity learning rules.

## Correlation with retinal waves instructs axon branch addition and elimination

**Fig. 1.**
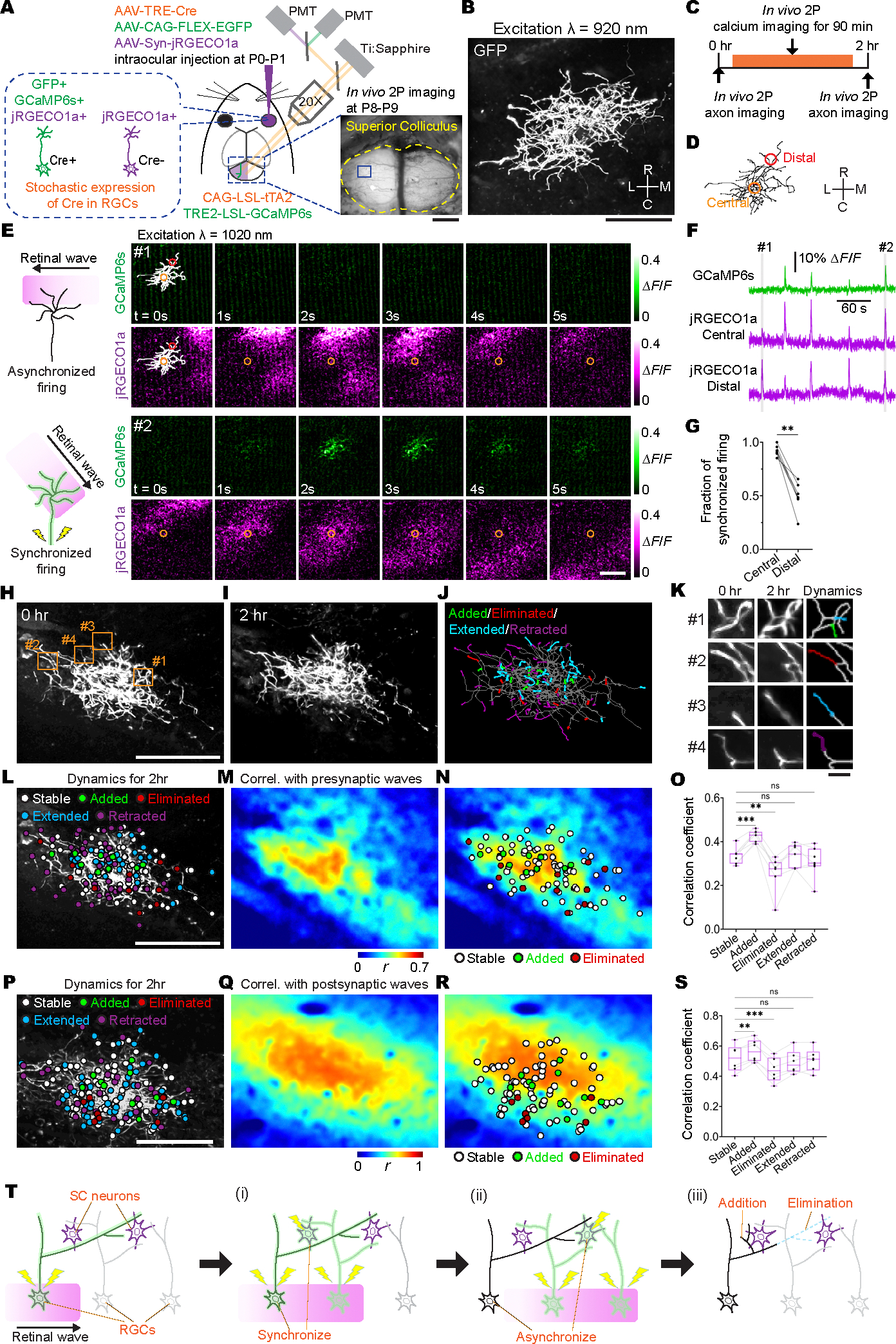
Synchronized firing between individual RGC axons and retinal waves instructs axon branch dynamics. (**A**) Schematic of the in vivo two-photon (2P) imaging approach for tracking axon branch dynamics of a single RGC and simultaneous dual-color calcium imaging of the RGC axon activity and retinal waves in the SC of awake, head-restrained mice at P8 to P9. Stochastic expression of Cre in a few RGCs was achieved by intravitreal injections of AAV2/1-TRE-Cre in Ai162 mice, which harbor CAG-LSL-tTA2 and TRE2-LSL-GCaMP6s alleles. AAV2/2-CAG-FLEX-EGFP and AAV2/1-Syn-jRGECO1a were also injected intravitreally. As a result, Cre^+^ RGCs expressed GFP, GCaMP6s, and jRGECO1a, whereas Cre^−^ RGCs only expressed jRGECO1a. The blue-boxed area is magnified in (B). Scale bar, 500 μm. PMT, photomultiplier tube; Ti:Sapphire, Ti:Sapphire laser. (**B**) In vivo two-photon imaging of an entire single RGC axon within a depth of 200 μm below the surface of the SC at P8. Directions R, L, M, and C correspond to rostral, lateral, medial, and caudal in the SC, respectively, unless otherwise stated. Scale bar, 100 μm. (**C**) Experimental timeline. Z stacks of a single–RGC axon arbor were acquired at a 2-hour interval, and dual-color calcium imaging was performed for 90 min in between taking the z stacks. (**D**) Traced z projection of axon branches from a single RGC within ±20 μm of the optical plane for dual-color calcium imaging. Orange and red circles indicate central and distal regions of interest (ROIs) for calculating the retinal wave traces shown in (F), respectively. (**E**) Example ΔF/F (fractional change in fluorescence) montages of single-axon firing (GCaMP6s) and of retinal waves (jRGECO1a). In montage 1, the axon did not fire when a retinal wave partially overlapped with its axon arbors. In montage 2, the axon fired when a retinal wave passed the center of the axon arbor. Orange and red circles indicate ROIs in the central and distal regions of the axon, respectively. Scale bar, 100 μm. (**F**) A GCaMP6s signal trace (ΔF/F) from the single RGC axon and jRGECO1a signal traces (ΔF/F) from the ROIs indicated in (D). The periods (1 and 2) depicted in montages (E) are shown in gray. (**G**) The fraction of synchronization between single-axon firing and retinal waves at central regions of the axons was significantly higher than that at distal regions. Data points from the same axon were paired (central, 0.92 ± 0.02; distal, 0.49 ± 0.05; **P = 0.008, one-tailed Wilcoxon signed-rank test, *n* = 7 axons from 7 animals). (**H** and **I**) Z projection of a single RGC axon at 0 (H) and 2 hours (I) of in vivo two-photon imaging. The orange-boxed areas in (H) are magnified in (K). Scale bar, 100 μm. (**J**) A single reconstructed RGC axon. The behavior of individual branches over the 2-hour interval were categorized as added (green), eliminated (red), extended (cyan), and retracted (magenta). (**K**) Zoomed-in images [corresponding to numbered orange boxes in (H)] show changes of axon branches over the 2-hour imaging session. The left and middle columns show projected images of two to three optical sections with 2-μm intervals at 0 and 2 hours. The right column displays changes of branch dynamics in 2 hours. Scale bar, 10 μm. (**L**) Terminals of stable (white), eliminated (red), extended (cyan), and retracted (magenta) axon branches at 2 hours were labeled on a z projection of the single–RGC axon arbor. The positions from which newly added branches emerged were also labeled (green). Scale bar, 100 μm. (**M**) A spatial map of Pearson’s correlation coefficients (*r*) between the activity of the single RGC axon and retinal waves. (*N*) Stable and eliminated branch terminals and positions where newly added branches emerged from were plotted on the correlation map. (**O**) Means of correlation coefficients [stable, 0.34 ± 0.01; added, 0.42 ± 0.01, *P* = 0.0004; eliminated, 0.26 ± 0.03, *P* = 0.001; extended, 0.33 ± 0.02, *P* = 0.999; retracted, 0.31 ± 0.03, *P* = 0.36; ns, not significant; one-way analysis of variance (ANOVA) with Dunnett’s multiple comparison test, *n* = 7 axons from 7 animals]. (**P** to **S**) Simultaneous in vivo two-photon imaging of single–RGC axon branch dynamics and dual-color calcium imaging of axonal activity and postsynaptic waves in the SC. Stochastic expression of Cre in a few RGCs was achieved by intravitreal injections of AAV2/1-TRE-Cre in Ai162 mice, which harbor CAG-LSL-tTA2 and TRE-LSL-GCaMP6s alleles, and expression of jRGECO1a in neurons of the SC was achieved by injection of AAV2/9-Syn-NES-jRGECO1a into the SC one day after eye injection. After the AAV injections, only a few RGC axons expressed GFP and GCaMP6, and most of SC neurons expressed jRGECO1a. (P) Terminals of stable, eliminated, extended, and retracted axon branches at 2 hours were labeled on a z projection of the single RGC axon arbor. The positions from which newly added branches emerged were also labeled. Scale bar, 100 mm. (Q) A spatial map of Pearson’s correlation coefficients between presynaptic activity of the single–RGC axon arbor and postsynaptic waves. (R) Stable and eliminated branch terminals and positions from where newly added branches emerged were plotted on the correlation map. (S) Mean correlation coefficients (stable, 0.52 ± 0.03; added, 0.57 ± 0.03, *P* = 0.0097; eliminated, 0.44 ± 0.03, *P* = 0.0002; extended, 0.49 ± 0.03, *P* = 0.29; retracted, 0.51 ± 0.03, *P* = 0.88, one-way ANOVA with Dunnett’s multiple comparison test, *n* = 6 axons from 6 animals). (**T**) Schematic model of Hebbian axon remodeling by endogenous patterns of spontaneous activity. Synchronized presynaptic inputs (retinal waves) excite postsynaptic cells (i and ii). When an RGC axon takes part in firing a SC neuron repeatedly or persistently (i), the axon forms new branches near the SC neurons to increase their efficacy (iii). By contrast, when an RGC axon does not participate in making an SC neuron fire repeatedly or persistently (ii), branches of the axon near the SC neuron are eliminated (iii). For all the box plots, the central line indicates the median, and the bottom and top edges indicate the 25th and 75th percentiles of the data across animals, respectively. **P < 0.01; ***P < 0.001; ns, not significant. Data are mean ± SEM.

To investigate the spatiotemporal relationship between RGC axon branch dynamics and retinal waves in the mouse superior colliculus (SC), we established a simultaneous in vivo two-photon imaging method combining time-lapse imaging of a single RGC axon with dual-color calcium imaging of both single–RGC axon activity and retinal waves (Fig. 1A). This proved technically challenging because of the need for bright and sparse labeling of axons, optical accessibility, high spatial resolution, and a method to resolve both the activity in a single axon and the ensemble activity of the retinal wave. To label RGC axons sparsely and brightly, we exploited leaky Cre recombinase expression driven by the tetracycline response element (TRE) promoter in the absence of tetracycline transactivator (tTA) and coupled it with Cre-dependent tTA-TRE–based positive-feedback amplification in the Supernova system (36). We injected adeno-associated virus (AAV)–TRE-Cre, AAV-Syn-jRGECO1a (a red-shifted genetically encoded calcium indicator), and AAV-FLEX-CAG–enhanced green fluorescent protein (EGFP) into the ocular vitreous of Ai162 mice, which express Cre-dependent TRE-GCaMP6s (a genetically encoded calcium indicator) and Cre-dependent tTA (37). This method resulted in only a few RGCs expressing EGFP and GCaMP6s, whereas most RGCs expressed jRGECO1a. Using in vivo two-photon imaging, we simultaneously recorded single–RGC axon branch dynamics within intertwined neighboring axon arbors in the SC (through EGFP fluorescence), axon firing (through GCaMP6s fluorescence) of the same EGFP-expressing single RGC axons, and retinal waves (through jRGECO1a fluorescence) propagating through dense RGC axon populations in the SC of awake, behaving mice during a transient window of development at postnatal days 8 to 9 (P8 to P9) (Fig. 1, B and C; fig. S1, A to G; and movie S1).

It has been challenging to distinguish the activity of individual axons amongst the synchronized spontaneous firing of neighboring axons in vivo owing to the technical difficulty of isolating single-axon firing from the bulk activity of many neighbors (38). Using in vivo dual-color calcium imaging of GCaMP6s in single RGC axons and jRGECO1a in bulk-labeled RGC axons, we measured the correlation between individual RGC axon firing and retinal waves in the SC. Notably, we found that the timing of individual axon firing was well regulated: Single-axon firing was highly synchronous with retinal waves when the wave propagated through the center of the axon arbor, but the fraction of synchronized firing was significantly reduced when retinal waves only passed through peripheral axon branches (Fig. 1, D to G, and movie S2). Because retinal waves are formed by synchronized firing among neighboring RGCs (39), this result suggests that the correlation of activity between an axon branch and neighboring axons is not uniform across the arbor of a single RGC (Fig. 1M). Notably, the degree of activity synchronization was related to where an axon branch was added or eliminated in the RGC axon arbor (Fig. 1, H to O, and fig. S2). Because retinal wave patterns were highly synchronized along the depth of the upper stratum griseum superficiale (SGS) of the SC (fig. S1H and movie S3), we could calculate the correlation between the firing of individual axon branches and retinal waves throughout an 80-μm stack by measuring single-axon firing and retinal waves in a single-image plane in the middle of the stack. We then quantified the relationship between the activity correlation and axon branch dynamics as revealed by time-lapse imaging of individual RGC axon morphology in the upper SGS (Fig. 1, H to K, and movie S4). We found that newly added branches were often located in regions where axon firing was highly synchronized with retinal waves propagating through neighboring axons, whereas eliminated branches were found in regions where axon firing was relatively asynchronous with waves (Fig. 1, N and O, and fig. S2). We also observed that added and eliminated branches were preferentially distributed in the central and distal parts of their axon arbors, respectively, though the position of added and eliminated branches was more related to the branches’ correlation with retinal waves than their distance from the center of the axon arbor (figs. S2 and S3). These observations suggest that the activity correlation with retinal waves instructs individual RGC axon connectivity at the resolution of single-axon branches.

By using one-photon wide-field dual-color calcium imaging, a recent study demonstrated that bulk retinal wave activity in presynaptic retinal axons and postsynaptic SC neurons was highly synchronized (19). In this study, using two-photon dual-color calcium imaging of single–RGC axon firing and SC neuron activity (fig. S4A), we observed a high level of synchrony between the firing of individual RGC axons and the wave activity of neighboring SC neurons at the center of the RGC axon arbors but not at distal arbors (fig. S4, B to D, and movie S5). Our data also shows that added and eliminated RGC axon branches were found in regions where axon firing was relatively synchronous and asynchronous with postsynaptic activity, respectively (Fig. 1, P to S). Notably, this is consistent with Hebb’s original prediction that “when an axon of cell A is near enough to excite a cell B and repeatedly or persistently takes part in firing it, some growth process or metabolic change takes place in one or both cells such that A’s efficiency, as one of the cells firing B, is increased” (13). It has long been hypothesized that sculpting the morphology of single terminal arbors through the addition and stabilization of branches in the vicinity of appropriate partners is required for making future synaptic contacts, which in turn increases the efficacy of exciting appropriate postsynaptic partners (Fig. 1T) (1, 40). Our data provides the first in vivo observation of this phenomenon, suggesting that endogenously generated activity regulates circuit connectivity at the single-axon level following a Hebbian learning rule.

## Decoupling axon activity from retinal waves randomizes branch addition and elimination

**Fig. 2.**
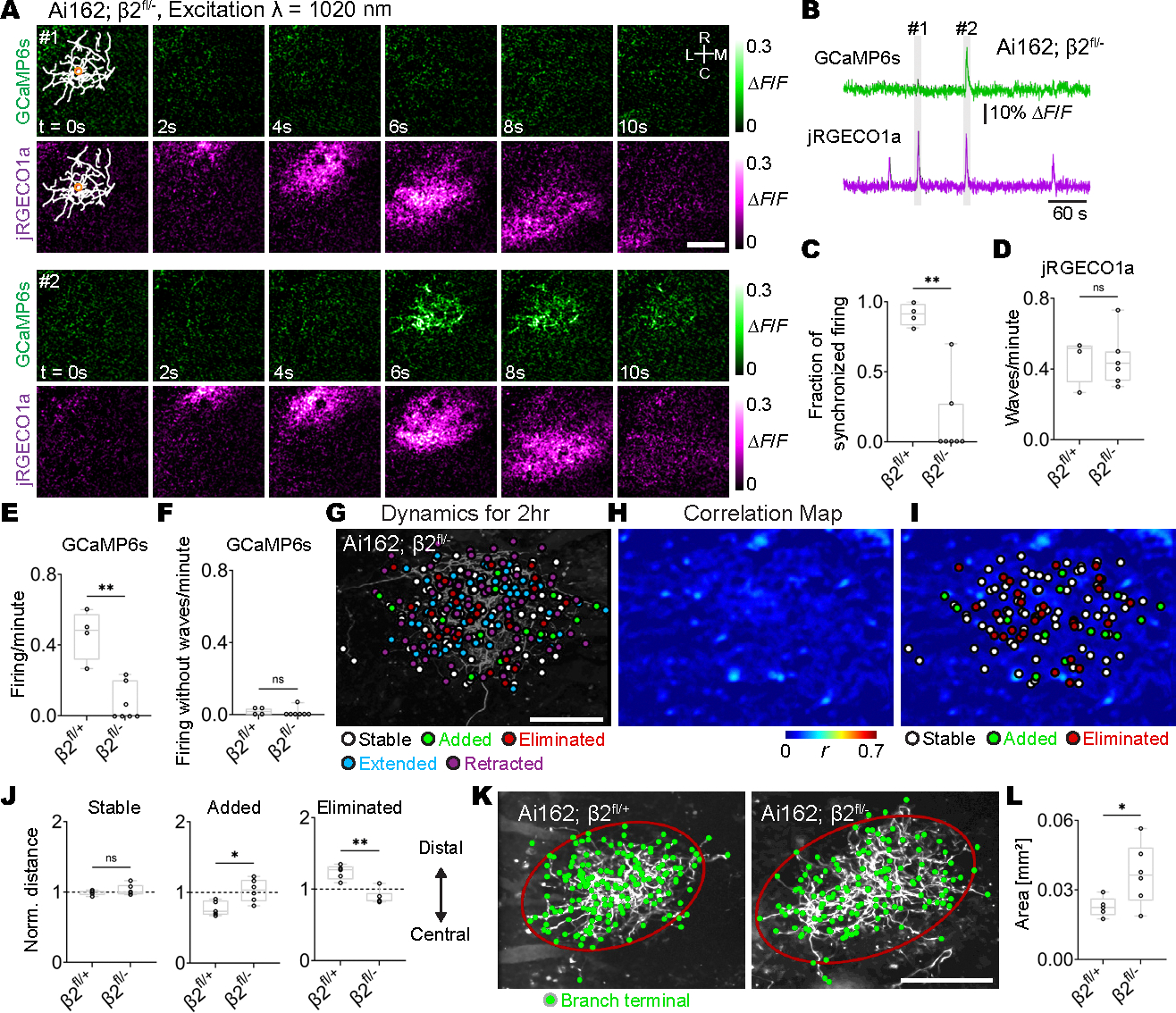
Decoupling synchronization between retinal waves and individual axon firing results in random distribution of added and eliminated axon branches. β2-nAChR was knocked out in only a few RGCs by intravitreal injections of AAV2/1-TRE-Cre in Ai162; β2^fl/−^ mice. (**A**) ΔF/F montages of single-axon firing (GCaMP6s) and of retinal waves (jRGECO1a) in Ai162; β2^fl/−^. In montage 1, a β2-nAChR–knockout axon did not fire when a retinal wave covered the center of the axon arbor, but it did fire in montage 2. Orange circles correspond to a ROI in the central region of the axon for traces in (B). Scale bars, 100 μm. (**B**) GCaMP6s and jRGECO1a signal traces (ΔF/F) of the ROI indicated in (A) from an Ai162; β2^fl/−^ mouse. The periods (1 and 2) depicted in montages (A) are shown in gray. (**C**) The fraction of retinal waves and single-axon firing that are synchronized in β2-nAChR–knockout (Ai162; β2^fl/−^) and control axons (Ai162; β2^fl/+^) (β2^fl/+^, 0.91 ± 0.03; β2^fl/−^, 0.14 ± 0.09; **P = 0.003, one-tailed Wilcoxon rank sum test). (**D**) The frequency of retinal waves was not altered (β2^fl/+^, 0.45 ± 0.06; β2^fl/−^, 0.45 ± 0.05; *P* = 0.28, one-tailed Wilcoxon rank sum test). (**E**) The frequency of spontaneous firing was reduced in β2-nAChR–knockout axons (β2^fl/+^, 0.46 ± 0.06; β2^fl/−^, 0.07 ± 0.04; *P* = 0.003, one-tailed Wilcoxon rank sum test). (**F**) The frequency of single-axon firing in the absence of retinal waves was not altered (β2^fl/+^, 0.017 ± 0.008; β2^fl/−^, 0.010 ± 0.009; *P* = 0.28, one-tailed Wilcoxon rank sum test). [(C) to (F)] β2^fl/+^, *n* = 4 axons from 4 animals at P8; β2^fl/−^, *n* = 7 axons from 7 animals at P8. (**G**) Terminals of stable (white), eliminated (red), extended (cyan), and retracted (magenta) axon branches at 2 hours were labeled on a z projection of a β2-nAChR–knockout RGC axon arbor. The positions from which newly added branches emerged were also plotted (green). Scale bar, 100 μm. (**H**) A spatial map of Pearson’s correlation coefficient between the firing of the β2-nAChR–knockout RGC axon and retinal waves. (**I**) Stable and eliminated branch terminals and the positions from which added branches emerged were plotted on the correlation map. (**J**) Distances from the center of single-axon arbors to their stable and eliminated branch terminals and added branch points. Distances were normalized by the average distance from the center to each branch. The normalized values would be one if axon branch positions were randomly distributed and would be smaller than one if axon branch positions were distributed near the center (stable: β2^fl/+^, 1.00 ± 0.02; β2^fl/−^, 1.03 ± 0.03; *P* = 0.47. added: β2^fl/+^, 0.78 ± 0.04; β2^fl/−^, 1.03 ± 0.06; *P* = 0.015. eliminated: β2^fl/+^, 1.24 ± 0.04; β2^fl/−^, 0.89 ± 0.04; *P* = 0.002. β2^fl/+^, *n* = 5 axons from 5 animals at P8; β2^fl/−^, n = 6 axons from 6 animals at P8; one-tailed Wilcoxon rank sum test). (**K**) Axon branch terminals (green) and covariance ellipses with a 90% confidence interval (red) were overlaid on the z projection of a single RGC axon from an Ai162; β2^fl/+^ control mouse (left) and from an Ai162; β2^fl/−^ mouse (right) at P8. Scale bar, 100 μm. (L) The areas of covariance ellipses were enlarged in β2-nAChR–knockout axons (β2^fl/+^, 0.023 ± 0.002 mm^2^, *n* = 5 axons from 5 animals at P8; β2^fl/−^, 0.037 ± 0.005 mm^2^, *n* = 6 axons from 6 animals at P8; *P* = 0.04; one-tailed Wilcoxon rank sum test). *P < 0.05; **P < 0.01; ***P < 0.001; ns, not significant. Data are mean ± SEM.

We next tested the effect of decoupling individual RGC activity from retinal waves on axon arbor dynamics. Earlier studies demonstrated that stage 2 retinal waves (P8 to P9) depend on cholinergic neurotransmission mediated by nicotinic acetylcholine receptors (nAChRs) containing β2 subunits (7, 41). Genetic ablation of β2-nAChRs results in severe disruption of retinal waves (23, 42). To decouple individual RGC activity and retinal waves, we knocked out β2 subunits selectively in sparsely labeled RGCs by taking advantage of our modified Supernova system in which only a few RGCs expressed Cre in Ai162; floxed β2-nAChR (Ai162; β2^fl/−^) mice (Fig. 2, A to F). Dual-color calcium imaging at P8 showed that the activity of individual β2-knockout axons was no longer synchronized with retinal waves even when measured at the center of the axon arbor (Fig. 2, B and C, and movie S6). Although knocking out β2 subunits in a small number of RGC axons in Ai162; β2^fl/−^ mice did not affect the overall frequency of retinal waves in comparison to that of control mice (Ai162; β2^fl/+^) (Fig. 2D), the firing of β2-knockout axons was drastically reduced (Fig. 2E). This finding reinforces the importance of β2-nAChRs in RGCs for participation in retinal waves during development (7, 41). In control mice, single-axon firing rarely occurred in the absence of retinal waves passing through the center of its axon arbor (Fig. 2F). This was also true of β2-knockout axons (Fig. 2F), indicating that the decreased fraction of synchronized firing in β2-knockout axons (Fig. 2C) is not due to the increase in the firing of β2-knockout axons without retinal waves. Notably, the synchrony between axon firing of β2-knockout axons and retinal waves was greatly reduced and no longer different when the wave propagated through the center of the axon arbor and when retinal waves only passed through peripheral axon branches (fig. S5A). Consistent with this, decoupling synchronized firing between β2-knockout axons and retinal waves resulted in random positioning of added and eliminated axon branches (Fig. 2, G to L). In control mice, added and eliminated branches were preferentially distributed in the central and distal parts of the RGC axon arbor, respectively (fig. S5B), whereas preferential axon branch addition and elimination was absent in β2-knockout axons from Ai162; β2^fl/−^ mice (Fig. 2J and fig. S5C). Moreover, the area of the SC innervated by individual β2-knockout axons was significantly increased in Ai162; β2^fl/−^ mice (Fig. 2, K and L), consistent with previous studies using whole-animalβ2^−/−^ mice (28, 30). These results demonstrate that, in the absence of instruction by retinal waves, terminal branches of individual RGC axon arbors lose their target specificity.

## Axon arbors extend in similar directions as retinal wave correlations

**Fig. 3.**
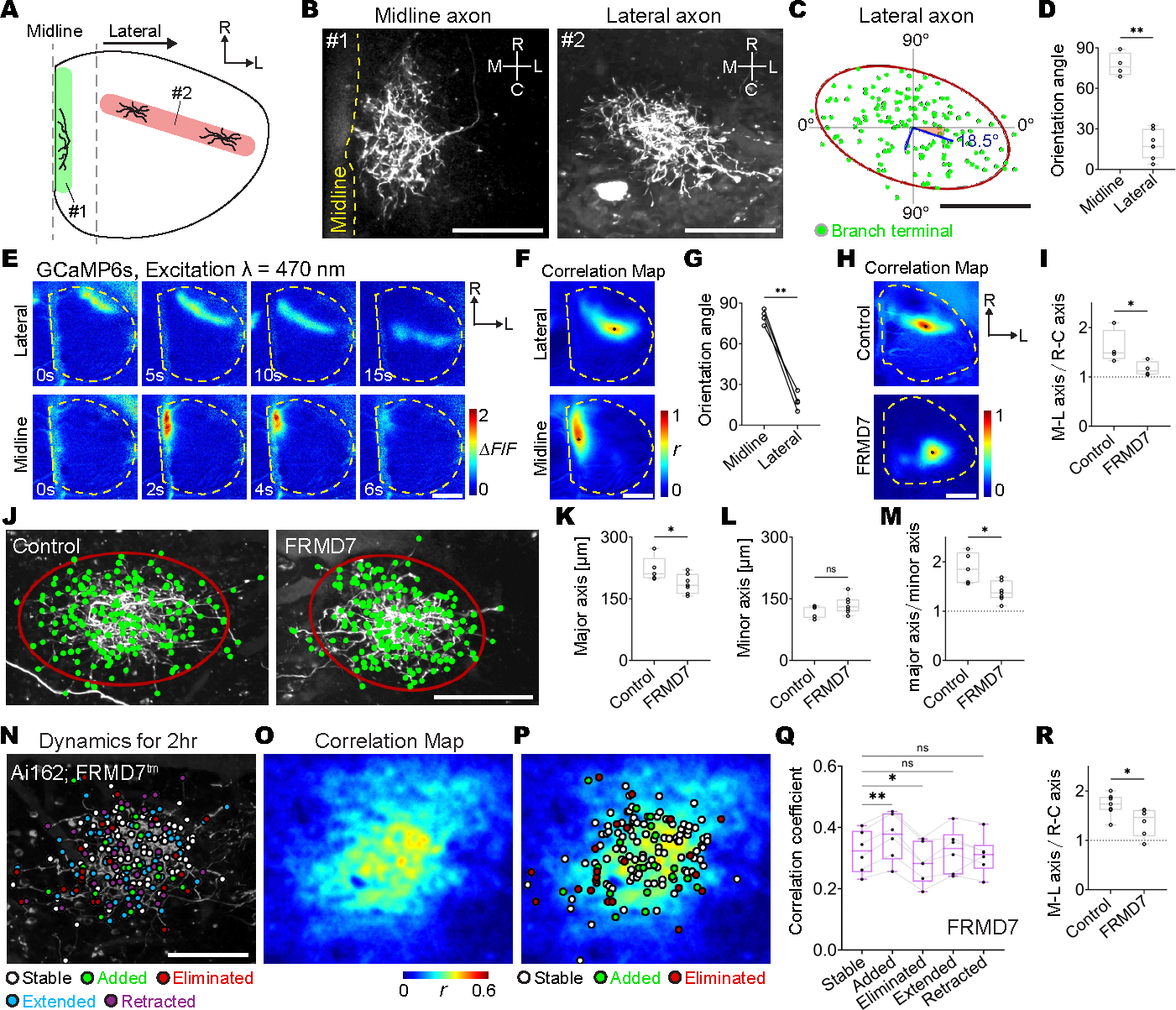
Patterns of retinal waves are related to the direction of RGC axonal extension. (**A**) Schematic illustration of distinct directions of RGC axonal extension in the midline region (1, green; within 200 μm from the midline) and the lateral region (2, red; at least 400 μm away from the midline) of the SC. (**B**) Z projections of single RGC axons with rostrocaudal extension in the midline region (1) and with mediolateral extension in the lateral region (2). Scale bars, 100 μm. (**C**) Ellipse fitting for a mediolaterally extended RGC axon in the lateral region of the SC. A covariance ellipse with a 90% confidence interval (red) was calculated from axon branch terminal positions (green) by using principal component analysis (PCA). The eigenvectors with the length corresponding to the eigenvalues are also shown (blue). Scale bar, 100 μm. (**D**) Orientation angles of the major axes of covariance ellipses at P8 to P9 (midline region, 77.4 ± 3.8°, *n* = 4 axons from 4 animals; lateral region, 18.7 ± 3.7°, *n* = 7 axons from 7 animals; *P* = 0.003, one-tailed Wilcoxon rank sum test). (**E** to **G**) In vivo wide-field single-photon calcium imaging of retinal waves in the SC at P8 to P9. GCaMP6s expression in RGCs was achieved by intravitreal injections of AAV2/1-Syn-GCaMP6s at P0 to P1. (E) ΔF/F montages of a retinal wave propagating in the lateral region (top) and of flashlike spontaneous activity in the midline area (bottom). Scale bar, 500 μm. (F) Examples of seed-based correlation maps of retinal waves, with the seed located in the lateral (top) and the midline regions (bottom), respectively. Black dots indicate seed locations. Scale bar, 500 μm. (G) Orientation angles of the area where correlation coefficient values in a correlation map were above the threshold (4 SD + mean) (midline, 80.0 ± 2.3°; lateral, 17.7 ± 2.7°; *P* = 0.001, one-tailed Wilcoxon signed-rank test, *n* = 4 animals at P9). (**H** and **I**) In vivo wide-field single-photon calcium imaging of RGC axons in the SC of FRMD7^tm^ mice. (H) Examples of seed-based correlation maps when seed locations were in the lateral region of the SC in a control mouse (top) and in a FRMD7 mutant mouse (bottom). Black dots indicate seed locations. Scale bar, 500 μm. (I) The ratio of the mediolateral axis length over the rostrocaudal axis length for the high correlation area was reduced in FRMD7 mutant mice (control, 1.58 ± 0.15, *n* = 4 animals at P9; FRMD7, 1.18 ± 0.04, *n* = 4 animals at P9; *P* = 0.03, one-tailed Wilcoxon rank sum test). A ratio of more than 1 suggests that the high correlation area is mediolaterally extended. (**J** to **M**) Single-axon morphology in FRMD7^tm^ mice. Sparse GFP expression in RGCs was achieved by intravitreal injections of AAV2/1-TRE-Cre and AAV2/2-CAG-FLEX-EGFP in Ai162; FRMD7^tm^ mice. (J) Axon branch terminals (green) and their covariance ellipses with a 90% confidence interval (red) were plotted over the z projections of a single RGC axon in Ai162; FRMD7^tm^ (right) and its control (left) at P9. Scale bar, 100 μm. (K) Lengths of the major axes of covariance ellipses with a 90% confidence interval calculated from axon branch terminal positions of individual axon arbors (control, 221.9 ± 12.1 μm; FRMD7, 186.5 ± 8.2 μm; *P* = 0.024, one-tailed Wilcoxon rank sum test). (L) Lengths of minor axes of covariance ellipse (control, 119.6 ± 5.9 μm; FRMD7, 135.3 ± 7.6 μm; *P* = 0.13, one-tailed Wilcoxon rank sum test). (M) The ratio of the length of the major axis over the length of the minor axis of the covariance ellipse (control, 1.88 ± 0.13; FRMD7, 1.40 ± 0.07; *P* = 0.015, one-tailed Wilcoxon rank sum test). [(K) to (M)] All axons were from lateral regions. Control, *n* = 5 axons from 5 animals at P9; FRMD7, *n* = 7 axons from 7 animals at P9. (**N**) Terminals of stable (white), eliminated (red), extended (cyan), and retracted (magenta) axon branches at 2 hours were labeled on a z projection of a single–RGC axon arbor in an Ai162; FRMD7^tm^ mouse. Positions from which newly added branches emerged were also plotted (green). Scale bar, 100 μm. (**O**) A spatial map of Pearson’s correlation coefficients between the activity of a single RGC axon and retinal waves in a FRMD7 mutant. (**P**) Stable and eliminated branch terminals and positions from which added branches emerged were plotted on the correlation map. (**Q**) Mean correlation coefficients for axon branches exhibiting different dynamics were significantly different in FRMD7 mutant mice (stable, 0.32 ± 0.03; added, 0.37 ± 0.03, *P* = 0.002; eliminated, 0.28 ± 0.03, *P* = 0.016; extended, 0.32 ± 0.03, *P* = 0.999; retracted, 0.31 ± 0.02, *P* = 0.682; one-way ANOVA with Dunnett’s multiple comparison test, *n* = 6 axons from 6 animals at P9). (**R**) The ratio of the mediolateral axis length over the rostrocaudal axis length for area with correlation coefficients of more than 2.5 SD + mean in a spatial map of Pearson’s correlation coefficients between the activity of a single RGC axon and retinal waves (control, 1.72 ± 0.08, *n* = 7 axons from 7 animals; FRMD7, 1.37 ± 0.11, *n* = 6 axons from 6 animals; *P* = 0.01, one-tailed Wilcoxon rank sum test). A ratio >1 suggests that the high correlation area is mediolaterally extended. *P < 0.05, **P < 0.01. Data are mean ± SEM.

Notably, the shape of individual RGC axon arbors was related to the position of the axon in the SC (Fig. 3A). In particular, individual RGC axon arbors near the midline were rostrocaudally extended, consistent with a previous study using bulk axon labeling methods (43), whereas RGC axon arbors in the lateral region of the SC were mediolaterally oriented (Fig. 3, B to D). The selective extension of RGC axon arbors also suggests that the position of added and eliminated branches were not determined simply by the distance of the branch from the center of the axon arbors: If branch position depended solely on distance from the center of the axon, then the terminal zone of single axons should be circular. Moreover, the orientation of RGC axon arbors was also consistent with the spatial pattern of local correlations of retinal wave activity. Local correlations near the midline were rostrocaudally extended, whereas local correlations in the lateral region of the SC were mediolaterally extended (Fig. 3, E to G, and movie S7). To further investigate whether the pattern of retinal waves was associated with RGC axon morphology, we examined an FRMD7 mutant mouse, which has altered retinal wave patterns (10). FRMD7 is specifically expressed in retinal starburst amacrine cells, which are responsible for the pattern of retinal waves in the retina (44), and FRMD7 mutant mice have reduced temporal-to-nasal propagating waves in retinocollicular axons (or rostral-to-caudal waves in the SC) without changes in the size of individual wavefronts or the frequency of retinal waves (10). We found that in FRMD7 mutant mice, the mediolateral extent of local wave activity correlations, which represent accumulated wavefronts but not individual wavefronts during the imaging period, was reduced compared with that of control mice (Fig. 3, H and I, and movie S8). Furthermore, the mediolateral but not the rostrocaudal extension of RGC axon arbors was significantly decreased in FRMD7 mutant mice (Fig. 3, J to M), reinforcing the relationship between endogenous patterns of retinal waves and individual RGC axon morphology in the SC. We further observed that added and eliminated RGC axon branches in FRMD7 mutant mice were still found in regions where axon firing was relatively synchronous and asynchronous with retinal waves, respectively (Fig. 3, N to Q). We also found that the mediolateral extension in the correlation pattern between single-axon firing and retinal waves was disrupted in FRMD7 mutant mice (Fig. 3R). The decreased mediolateral extension of RGC axon arbors was consistent with the change in spatial pattern of activity correlations along the mediolateral axis in FRMD7 mutant mice. These results reinforce the instructive role of retinal wave pattern on the refinement of individual axon branches: When the pattern of retinal waves is altered in FRMD7 mutant mice, local patterns of activity synchrony are altered, and as axon refinement follows local activity synchrony, axon morphology is consequently altered.

## Hebbian synaptic plasticity underlies the instructive role of retinal waves

We investigated the mechanism underlying the instructive role of retinal waves in RGC axon branch dynamics. It is well known that synaptic plasticity follows Hebb’s law: Synchronous firing between pre- and postsynaptic cells stabilizes and strengthens their connecting synapses, whereas asynchronous firing decreases synaptic strength (13). We predicted that correlation patterns of retinal waves would instruct the anatomical positions of retinocollicular synapses based on Hebbian synaptic plasticity and that the presence or absence of a synapse further determines the positions of added and eliminated axon branches. Indeed, retinal waves seem to be sufficient to induce long-term potentiation on retinocollicular synapses, as previously reported in a study that paired stimulation between RGC axons and SC neurons to mimic retinal wave activity, resulting in retinocollicular synaptic strengthening through a Hebbian learning rule (45). Furthermore, previous studies reported that positions of added and eliminated branches were associated with positions of presynaptic sites: in *X*. *laevis*, RGC axon branches with mature presynaptic sites were more stable than those with immature presynaptic sites, and most new axon branches emerged near presynaptic sites (34, 46).

**Fig. 4.**
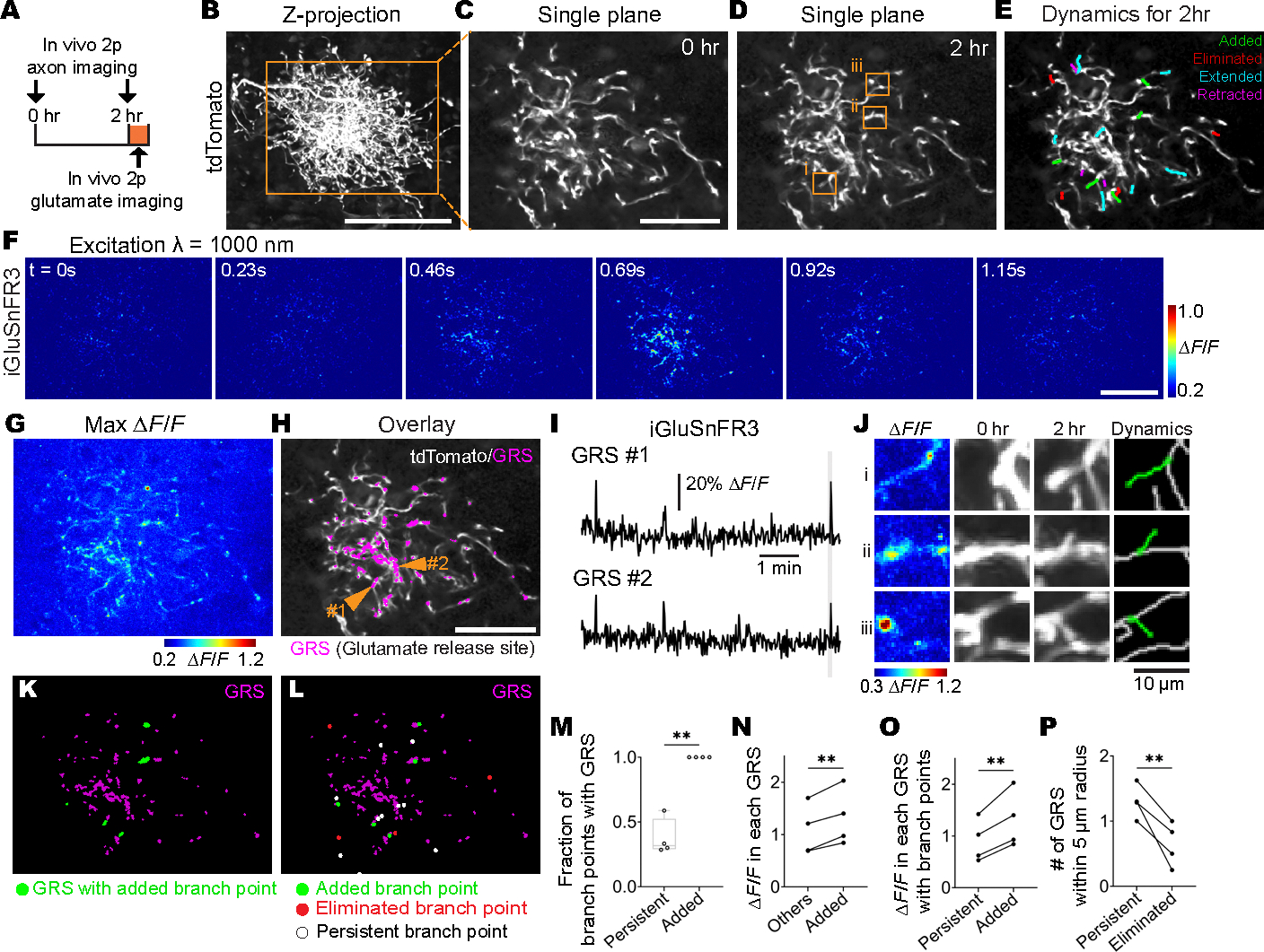
Presynaptic release sites are related to the position of added and eliminated branches at the single-axon level. In vivo two-photon imaging combining time-lapse imaging of axon branch dynamics and imaging of glutamate release from a single RGC in the SC of awake mice at P9. Stochastic expression of Cre in a few RGCs was achieved by intravitreal injections of AAV2/1-TRE-Cre and AAV2/2-CAG-FLEX-tTA2 in Ai9 mice, which harbor CAG-LSL-tdTomato. AAV2/1-hSyn-FLEX-iGluSnFR3-PDGFR was also injected intravitreally. As a result, only Cre^+^ RGCs expressed tdTomato and iGluSnFR3. (**A**) Experimental timeline. Z stacks of a single-RGC axon arbor were acquired at a 2-hour interval. In vivo two-photon glutamate imaging was performed for 6 min after taking the z stacks. (**B**) Z projection of a single RGC axon labeled by tdTomato and imaged in vivo at P9. The orange-boxed area is magnified in (C). Scale bar, 100 μm. (**C** and **D**) Optical sections of the single RGC axon at 0 (C) and 2 hours (D). The orange-boxed areas in (D) are magnified in (J). Scale bar, 100 μm. (**E**) The behavior of individual branches over the 2-hour interval was categorized as added (green), eliminated (red), extended (cyan), or retracted (magenta). (**F**) Example ΔF/F montages of single-axon glutamate release (iGluSnFR3). Scale bar, 100 μm. (**G**) Maximum values of ΔF/F of the example field of view. (**H**) GRSs (magenta) are overlaid on the optical section (white). GRSs were defined as axon segments with significant iGluSnFR signal (ΔF/F more than mean + 2 SD) and containing at least six pixels (1 μm^2^). (**I**) iGluSnFR signal traces (ΔF/F) corresponding to the GRSs indicated in (H). The period depicted in montages (F) are shown in gray. (**J**) Zoomed-in images corresponding to the numbered orange boxes in (D) that show changes of example axon branches over the 2-hour imaging session. (Left) Maximum values of ΔF/F (iGluSnFR3). (Middle) Projected images of three optical sections with 2-μm intervals at 0 and 2 hours (tdTomato). (Right) Added branches (green) in 2 hours. Scale bar, 10 μm. (**K**) GRSs with newly added branch points are shown in green. Scale bar, 100 μm. (**L**) The positions of added (green), eliminated (red), and persisting (white) branch points are overlaid on GRSs (magenta). (**M**) The fraction of branch points with GRS (persisting branch points, 0.38 ± 0.06; newly added branch points, 1.00 ± 0.00; *P* = 0.002, paired *t* test). (**N**) Means of ΔF/F were calculated from GRSs without added branch points (others, 1.1 ± 0.2) or from GRSs with newly added branch points (added, 1.3 ± 0.2) (*P* = 0.005, paired t test). (**O**) Means of ΔF/F were calculated from GRSs with persisting branch points (persisting, 0.9 ± 0.2) or from GRSs with newly added branch points (added, 1.3 ± 0.2) (*P* = 0.007, paired t test). (**P**) Means of numbers of GRSs within a 5-μm radius from persisting (persisting, 1.3 ± 0.1) or eliminated branch points (eliminated, 0.6 ± 0.1) (*P* = 0.009, paired *t* test). *n* = 4 axons from 4 animals. **P < 0.01. Data are mean ± SEM.

To investigate the relationship between the position of presynaptic terminals and branch dynamics at the single-axon level, we combined time-lapse imaging of a single RGC axon with in vivo two-photon imaging of presynaptic glutamate release in the same RGC axon. Utilizing the Supernova system, we achieved sparse labeling of only a few RGCs with tdTomato and the fluorescent glutamate indicator iGluSnFR3 (47). Following time-lapse imaging of single-axon morphology by tdTomato, we imaged spontaneous iGluSnFR3 signals in the same axon in the SC (Fig. 4, A to F). In vivo iGluSnFR3 imaging showed glutamate release sites (GRSs) on single axons in the SC (Fig. 4, F to I), suggesting that individual RGC axons formed synaptic contacts in the SC at the time of imaging. Moreover, the positions of newly added branches were highly associated with the position of glutamate release sites (Fig. 4, J to M), with 100.0 ± 0.0% of newly added branches adjacent to glutamate release sites. This was consistent with previous studies showing that new axon branches emerge at presynaptic sites labeled by GFP-tagged presynaptic proteins in RGC axons of zebrafish and *X. laevis* (34, 46, 48). Notably, although new branch points were always associated with glutamate release sites, only a small portion of glutamate release sites (7.7 ± 3.8%) were associated with branch formation. On the basis of previous experiments, it has not been possible to distinguish which presynaptic sites among many would contribute to branch formation (34, 46, 48). In this study, we observed that newly added branches were not random but preferentially located at regions with higher levels of glutamate release (Fig. 4N). Furthermore, among the many branch points, newly formed branch points had higher glutamate signals than persisting branch points (Fig. 4O). These results demonstrate that axon branches are preferentially added at relatively strong synaptic sites at the single-axon level, which would explain why only a few glutamate release sites among many contribute to branch addition (Fig. 4K). We further found that eliminated branches were often located in areas where glutamate release sites were relatively sparse (Fig. 4P), consistent with the idea that synaptic contacts locally stabilize branches (46, 48). Taken together, these results suggest that the positions of added and eliminated branches are strongly related to the positions of presynaptic sites on RGC axon arbors at the single-axon level.

**Fig. 5.**
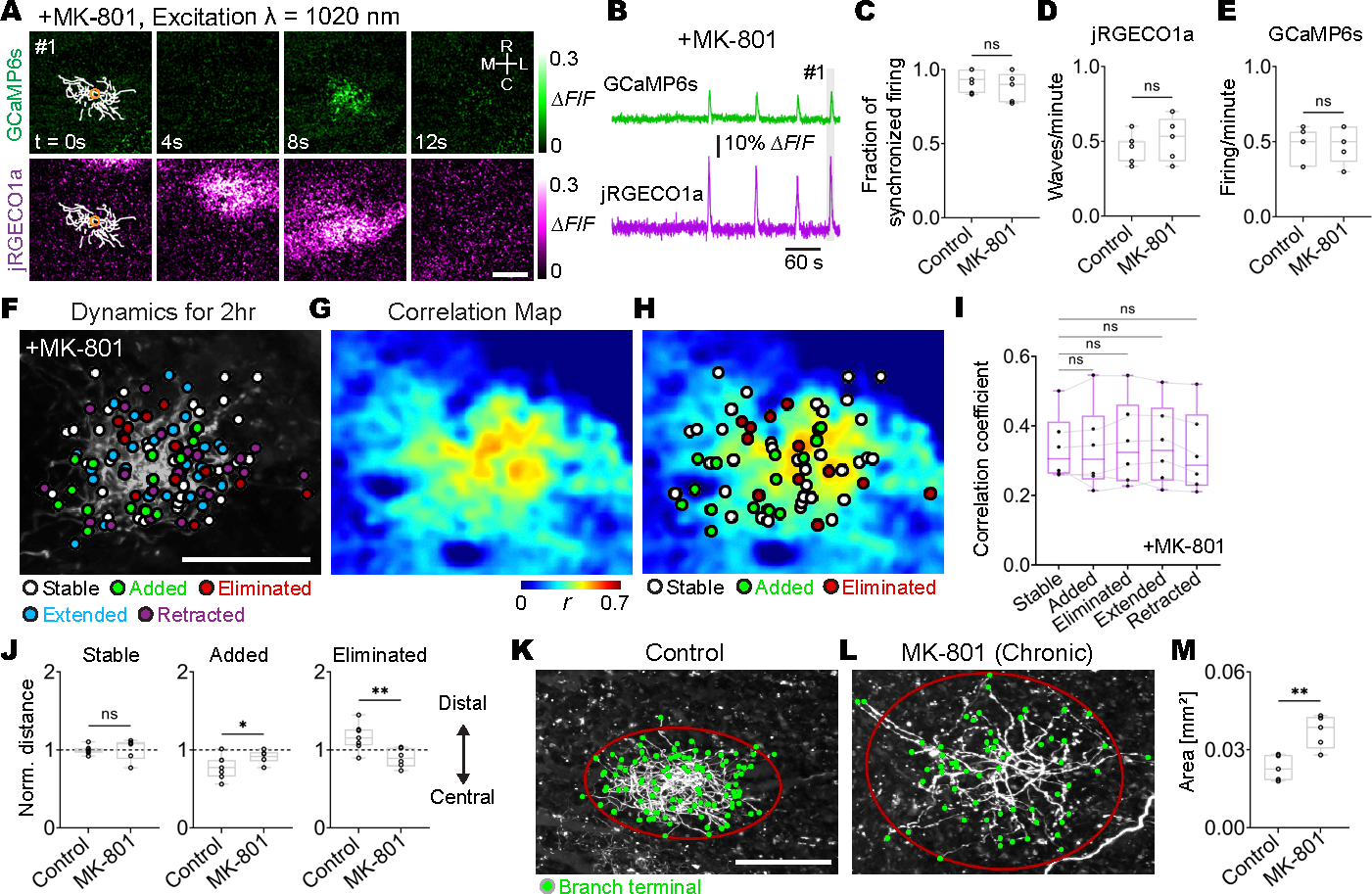
NMDAR is required for the instructive roles of retinal waves in axon branch dynamics. MK-801 was intraperitoneally injected into mice 30 min prior to imaging sessions. Z stacks of a single–RGC axon arbor were acquired at a 2-hour interval, and dual-color calcium imaging of single RGC axons (GCaMP6s) and retinal waves of bulk RGC axons (jRGECO1a) was performed for 90 min in between taking the z stacks. (**A**) ΔF/F montage of single-axon firing (GCaMP6s) and of a retinal wave (jRGECO1a) after MK-801 injection. Orange circles correspond to a central ROI for traces in (B). Scale bar, 100 μm. (**B**) GCaMP6s and jRGECO1a signal traces (ΔF/F) corresponding to the ROIs in (A). Gray area (1) indicates the period depicted in the montage in (A). (**C**) The fraction of retinal waves and single-axon firing that were synchronized in MK-801–treated and control mice (control, 0.93 ± 0.03; MK-801, 0.88 ± 0.04; *P* = 0.18, one-tailed Wilcoxon rank sum test). (**D**) Frequency of retinal waves (control, 0.47 ± 0.03; MK-801, 0.51 ± 0.06; *P* = 0.24, one-tailed Wilcoxon rank sum test). (**E**) Frequency of single-axon firing (control, 0.48 ± 0.04; MK-801, 0.49 ± 0.05; *P* = 0.44, one-tailed Wilcoxon rank sum test). [(C) to (E)] Control, *n* = 5 axons from 5 animals at P8 to P9; MK-801, *n* = 7 axons from 7 animals at P8 to P9. (**F**) Axon branch terminals of stable (white), eliminated (red), extended (cyan), and retracted (magenta) branches at 2 hours were labeled on a z projection of a single–RGC axon arbor in a MK-801–treated mouse. Positions from which newly added branches emerged were also plotted (green). Scale bar, 100 μm. (**G**) A spatial map of Pearson’s correlation coefficients between the activity of a single RGC axon and retinal waves. (**H**) Stable and eliminated branch terminals and positions from which added branches emerged were plotted on the correlation map. (**I**) Mean correlation coefficients for axon branches exhibiting different dynamics were not significantly different (stable, 0.34 ± 0.04; added, 0.34 ± 0.05, *P* = 0.99; eliminated, 0.35 ± 0.05, *P* = 0.61; extended, 0.35 ± 0.04, *P* = 0.78; retracted, 0.32 ± 0.04, *P* = 0.65; one-way ANOVA with Dunnett’s multiple comparison test, *n* = 6 axons from 6 MK-801–treated animals at P8 to P9). (**J**) Distances from the center of single axons to stable and eliminated branch terminals and to the points where new axon branches were added. Distances were normalized by the average distance from the center to each branch. Stable: control, 1.00 ± 0.02; MK-801, 1.01 ± 0.05; *P* = 0.27. Added: control, 0.78 ± 0.05; MK-801, 0.91 ± 0.03; *P* = 0.037. Eliminated: control, 1.16 ± 0.06; MK-801, 0.90 ± 0.04; *P* = 0.004. Control, *n* = 7 axons from 7 animals at P8 to P9; MK-801, *n* = 6 axons from 6 animals at P8 to P9; one-tailed Wilcoxon rank sum test. (**K** to **M**) MK-801 or saline (control) was intraperitoneally injected into mice every 24 hours from P5 to P8. Z stacks of single–RGC axon arbors were acquired at P8. Axon branch terminals (green) and covariance ellipses with a 90% confidence interval (red) were overlaid on the z projection of the single RGC axon from a mouse treated with saline (K) and from a mouse treated with MK-801 (L). Scale bar, 100 μm. (M) The areas of covariance ellipses were enlarged in axons of mice injected with MK-801 (control, 0.023 ± 0.002 mm^2^, *n* = 5 axons from 5 animals at P8; MK-801, 0.037 ± 0.003 mm^2^, *n* = 5 axons from 5 animals at P8; *P* = 0.008; one-tailed Wilcoxon rank sum test). *P < 0.05, **P < 0.01, ***P < 0.001. Data are mean ± SEM.

If retinal waves instruct the anatomical positions of retinocollicular synapses based on Hebbian plasticity rules, which in turn determines the positions of added and eliminated axon branches, then disruption of Hebbian synaptic plasticity would impair the instructive role of retinal waves in retinocollicular axon branch dynamics. Because *N*-methyl-d-aspartate receptors (NMDARs) act as molecular correlation detectors between pre- and postsynaptic activity, they have been repeatedly implicated in Hebbian learning and plasticity (49). We tested the effects of NMDAR blockade on RGC axon branch dynamics by performing intraperitoneal injections of the blood-brain barrier–permeable noncompetitive NMDAR antagonist MK-801. MK-801 did not alter the synchronization between individual axon firing and retinal waves in RGC axons (Fig. 5, A to C, and movie S9), nor did it lead to a decrease in individual axon firing or retinal wave frequency (Fig. 5, D and E). However, added and eliminated branches were found to be randomly distributed regardless of the spatial correlation pattern between axon firing and retinal waves in MK-801–treated mice (Fig. 5, F to I). The blockade of NMDARs also abolished the preferential addition of branches near the center of an axon arbor and the elimination of branches in the distal parts of axon arbors that were observed in controls (Fig. 5J and fig. S6, A and B). Further, chronic blockade of NMDARs resulted in enlarged terminal zones of single RGC axons in the SC (Fig. 5, K to M) and disrupted the mediolateral extension of RGC axon arbors in the lateral regions of the SC (fig. S7). Together, these results suggest that retinal waves instruct RGC axon branch dynamics through an NMDAR-dependent Hebbian mechanism.

## Discussion

Hebb’s rule of “fire together, wire together” serves as a basic organizing principle for models of learning and development. The results described here show that the fate of individual axon branches follows Hebb’s rule and depends on activity synchronization between individual retinal axons and SC neurons through spontaneous retinal waves: Axon branches are preferentially eliminated in arbor regions with low levels of local synchronization between single-axon firing and waves of spontaneous activity in neighboring neurons, whereas axon branches are added in regions with high levels of local synchronization. By using sparse conditional knockout of β2-nAChRs from RGC axons, we show that the instructive role of retinal waves in axon branch dynamics requires tight coupling between axon firing and retinal waves. Furthermore, by using glutamate imaging at the single-axon level, we show that the position of added and eliminated branches are highly correlated with the position of retinocollicular synapses. By blocking NMDARs with MK-801, we further show that retinal waves sculpt retinocollicular connectivity with subcellular precision through NMDAR-mediated Hebbian plasticity. These results indicate that retinal waves instruct the anatomical position of retinocollicular synapses through a Hebbian mechanism, which in turn determines the positions of added and eliminated axon branches at the single-axon level. Patterned spontaneous activity is found in many developing neural circuits, including the retina, cochlea, spinal cord, cerebellum, hippocampus, and neocortex, and is crucial for the development of proper connectivity patterns (7–9). Our findings provide fundamental insight into how endogenous patterns of correlated activity build precise neural circuits, confirming Hebbian principles in neural circuit development in vivo at the single-axon level.

For this study, we developed a distinct approach combining dual-color calcium imaging with single-axon labeling. This provided new insight into the patterns of synchronous firing between individual axons and their neighbors with high spatial resolution. Our in vivo dual-color calcium imaging revealed that individual RGC axons have heterogeneous spatial correlation maps between axon firing and retinal waves. This individualized instruction by retinal waves for each RGC axon might underlie the mechanism by which individual RGC axons establish their distinct connection patterns with appropriate partners in the SC. Our in vivo imaging approach also revealed the dynamic nature of axon branching in individual RGCs in awake mice. During a 2-hour observational window, axon arbors were highly dynamic with individual branches undergoing addition and elimination (figs. S5, D to G, and S6, C to F), which is difficult to appreciate based on snapshots of RGC axons over development in fixed samples (28). We also found that branch elimination was increased in single-cell β2-knockout RGC axons without changing the rate of branch additions, resulting in a decrease in the net number of axon branches during the imaging session (figs. S5 and S6). This likely also explains why the number of RGC axon branches is decreased in whole-animal β2^−/−^ mice (28). The results from single-cell β2-knockout RGC axons, including a decrease in the net number of axon branches and the random distribution of added and eliminated RGC axon branches (Fig. 2J), appear to be not due to the decrease in axon firing or activity-based competition because similar results were observed during the blockade of NMDARs (Fig. 5J), when the frequency of RGC axon firing was not significantly changed (Fig. 5E). Notably, presynaptic β2-nAchR–knockout axons rarely participated in retinal waves (Fig. 2, D to F), which resulted in asynchronous activity when postsynaptic cells were excited, but the β2-nAchR–knockout axons did not fire. Repeated postsynaptic firing without presynaptic firing might punish target specificity of individual RGC axon arbors.

Neighboring RGCs in the retina project their axons to neighboring neurons in the SC, thus preserving spatial information about visual space. Such retinotopic organization in the SC arises from two basic sources: the spatial arrangement of RGC axon projections in a given target region and the spatial extent of axonal convergence (22). Our in vivo imaging showed that the firing of individual RGC axons is highly synchronized with retinal waves at the center of the RGC axon arbors (Fig. 1G). This is likely because neighboring RGCs in the retina are highly synchronized during retinal waves, and these neighboring RGCs project their axons to largely overlapping regions in the SC. By contrast, distal RGC axon branches are likely situated in a region that is far away from the axon of neighboring RGCs in the retina, and therefore the distal branches and the surrounding RGC axons are less likely to be simultaneously recruited by retinal waves, resulting in asynchronous activity. We further observed that activity correlations between an axon branch and neighboring axons instructs where an axon branch is added or eliminated within the RGC axon arbor (Fig. 1, L to O). This axon remodeling mechanism helps to preserve the retinotopic spatial arrangement of RGC axons in the SC by eliminating unnecessary axon branches from a neighbors’ target area and promoting spatial refinement of receptive fields in target neurons. These data thus provide a direct in vivo demonstration of how retinotopic organization is refined by endogenous patterns of synchronized firing.

In mice, RGC subtypes are well characterized by their target depth in the SC: Terminal branches of J-, BD-, DRD4-, and W3-RGC subtypes were only found within 200-μm depth from the pial surface of the SC (upper SGS), whereas W7- and α-RGC subtypes send their axons to deeper SGS (30, 50, 51). A previous study showed that blockade of synchronized firing by β2 knockout results in the disruption of horizontal segregation but not the laminar distribution of α-RGC axons in the SC, suggesting that synchronized firing is important for horizontal extension rather than laminar segregation, which may be governed largely by molecular factors (30). In our system, we only focused on RGC axons whose terminal axon branches were within 200 μm of the pial surface of the SC (fig. S1D) and performed time-lapse imaging within 120 μm of the pial surface, where retinal waves are highly synchronized across depth (fig. S1H). Although multiple RGC subtypes could be included in our criteria, we did not find significant differences among randomly labeled axons in their synchronized firing with retinal waves (Figs. 1G, 2C, and 5C). Because the Supernova system is based on Cre-loxP recombination, we could not use transgenic Cre-driver mouse lines to distinguish RGC subtypes. Alternative sparse labeling and imaging strategies will be required for subtype specific analysis.

In our imaging system, we did not find differences in the correlation of axon firing and retinal waves among stable, extended, and retracted branches (Fig. 1O). Although we found weak correlations between the length of retraction and the activity correlation coefficient of axon firing and retinal waves (fig. S8), it is possible that these branches were part way through the elimination process during our imaging window. One plausible model is that branches randomly extend or retract to explore the environment (48), and once a branch makes synaptic contact with a partner, synaptic sites stabilize these branches and promote new branch formation (Fig. 4, M to P). This model is consistent with the “synaptotrophic hypothesis” that was proposed based on electron microscopic analysis (52). Further experiments are required to fully test these models.

Although our imaging system provides a powerful strategy to observe multiple in vivo events simultaneously, the imaging time window was limited: It is difficult to observe labeled RGC axons before P7 because of the random and often late onset of Cre expression. Late Cre expression also delayed the knockout of β2 subunits in labeled single RGC axons, enabling normal axon refinement for about a week after birth. This early refinement may give rise to the mediolateral extensions of axon arbors in lateral regions of the SC that we observed when we started imaging at P8. Further, it is difficult to image in vivo events after P10 because the SC is gradually covered by the neocortex at later ages. Alternative labeling and imaging strategies will be required for visualizing RGC axon development at earlier and later stages. Our ability to image glutamate signals both before and after time-lapse imaging of axon morphology was limited owing to photobleaching of tdTomato during glutamate imaging. New imaging strategies will be necessary to enable a more detailed and prolonged investigation of the relationship between the position of presynaptic sites and axon dynamics, including the extension and retraction of branches.

## Materials and methods summary

All animal procedures followed the Yale Institutional Animal Care and Use Committee (IACUC), the US Department of Health and Human Services, and institution guidelines. Mouse pups received intraocular or intracollicular injections of AAVs at P0 to P1 and were surgically implanted with a cranial window over the SC at P8 to P9 as previously described (10, 21). Stochastic expression of Cre in a few RGCs was achieved by intravitreal injections of AAV2/1-TRE-Cre in Ai162 mice, which harbor CAG-LSL-tTA2 and TRE2-LSL-GCaMP6s alleles, or AAV2/1-TRE-Cre and AAV2/2-CAG-FLEX-tTA2 in Ai9 mice, which harbor the CAG-LSL-tdTomato allele. β2-nAChR knockout in only a few RGCs was achieved by intravitreal injections of AAV2/1-TRE-Cre in Ai162; β2^fl/−^ mice. To alter retinal wave patterns, Ai162 mice were crossed with FRMD7 mutant mice. Calcium imaging and glutamate imaging were performed on unanesthetized, head-fixed mice in the dark. For in vivo two-photon dual-color calcium imaging, GCaMP6s and jRGECO1a were simultaneously excited at 1010 to 1020 nm. Z stacks of a single–RGC axon arbor were acquired at a 2-hour interval, and dual-color calcium imaging was performed for 90 min in between taking the z stacks. For acquiring z stacks of axon arbors, GFP and tdTomato were excited at 920 and 1000 nm, respectively. To reduce motion artifacts during the acquisition of z stacks, mice were briefly anesthetized with 1.5% isoflurane. For in vivo two-photon glutamate imaging, iGluSnFR3 was excited at 1000 nm. Movies for iGluSnFR3 were continuously acquired for 6 min in each recording session. Wide-field calcium imaging was performed as previously described (10). To block NMDAR activity, 15 μl of 1-mM MK-801 was intraperitoneally injected 30 min prior to imaging. For chronic treatments of MK-801, mice were intraperitoneally injected with 15 μl of 1-mM MK-801 or saline (control) every 24 hours from P5 to P8. Procedures for the experiments and data analyses are described in detail in the supplementary materials.

## Supplementary Material

Table S1

sMovie1

sMovie3

sMovie4

sMovie5

sMovie2

sMovie7

sMovie8

sMovie6

sMovie9

Supplementary

SUPPLEMENTARY MATERIALS

science.org/doi/10.1126/science.adh7814

## Data Availability

All data in the main text or the supplementary materials are available upon request to the corresponding author.
