# Supplementary material for: Hebbian instruction of axonal connectivity by endogenous correlated spontaneous activity
Source: Science. Author manuscript; Available in PMC 2025 Jun 9. (PMC12148345; doi:10.1126/science.adh7814)
Supplement: Supplementary [file NIHMS2078803-supplement-Supplementary.docx]

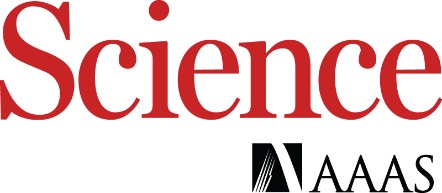


Supplementary Materials for

Hebbian instruction of axonal connectivity by endogenous correlated spontaneous activity

Naoyuki Matsumoto, Daniel Barson, Liang Liang, Michael C. Crair

Correspondence to: michael.crair@yale.edu (M.C.C.); liang.liang@yale.edu (L.L.)

**This PDF file includes:**

Materials and Methods

Figs. S1 to S8

Captions for Movies S1 to S9

Captions for Table S1

**Other Supplementary Materials for this manuscript include the following:**

Movies S1 to S9

Table S1

Materials and Methods

Animals

Animals of both sexes were used in this study. Animal care and use followed Yale Institutional Animal Care and Use Committee (IACUC) and US Department of Health and Human Services guidelines. Ai162(TIT2L-GC6s-ICL-tTA2)-D mice were obtained from the Allen Institute (*37*). SNAP25-GCaMP6s (stock number: 025111) and Ai9 (RCL-tdT)-D mice (stock number: 007909) were purchased from the Jackson Laboratory. Floxed β2-nAChR mice (β2^fl/fl^) and β2-nAChR null mice (β2^-/-^) have been described previously (*23*). Ai162; β2^fl/fl^ mice were crossed with Ai162; β2^+/-^ mice to obtain Ai162; β2^fl/-^ and control mice (Ai162; β2^fl/+^). Frmd7^tm1a(KOMP)Wtsi^ mice were provided by B. Roska (*44*). FRMD7^tm^ mice refer to the homozygous female or hemizygous male Frmd7^tm1a(KOMP)Wtsi^ mice. FRMD7^tm^ mice were crossed with Ai162 mice to obtain Ai162; FRMD7^tm^.

Eye injections

P0-P1 mice were anesthetized using hypothermia and were maintained on an ice pack for the duration of the procedure. For imaging single axon dynamics and calcium activity of single RGC axons and retinal waves, an AAV cocktail consisting of 400 nL of AAV2/2-pCAG-FLEX-GFP (#51502-AAV2, Addgene) (titer ≥ 7 × 10^12^ vg/ml), 400 nL of AAV2/1-Syn-NES-jRGECO1a (#100854-AAV1, Addgene) (titer ≥ 1 × 10^13^ vg/ml) and 30 nL of AAV2/1-TRE-Cre (#85040l, Addgene; packaged by Yale Vision Core) (titer ≥ 1 × 10^12^ vg/ml), was pressure injected into the eye through a pulled glass micropipette using a Nanoject (Drummond Scientific). Both right and left eyes were used in this study. After the injection, the pups were allowed to recover for 20 min on a heating pad before being returned to their mothers. For axon labeling with both GFP and tdTomato, P0 pups were injected with an AAV cocktail consisting of 400 nL of AAV2/2-pCAG-FLEX-GFP, 400 nL of AAV2/2-CAG-tdTomato (UNC) (titer ≥ 5.6 × 10^13^ vg/ml) and 30 nL of AAV2/1-TRE-Cre into the eye through a pulled glass micropipette using the Nanoject. For glutamate imaging, P0 pups of Ai9 mice were injected with an AAV cocktail consisting of 400 nL of AAV2/1-hSyn-FLEX-iGluSnFR3-v857-PDGFR (#175180-AAV1, Addgene) (titer ≥ 5 × 10^13^ vg/ml), 400 nL of AAV2/2-pCAG-FLEX2-tTA2-WPRE-bGHpA (#65458, Addgene; packaged by Yale Vision Core) (titer ≥ 5.5 × 10^13^ vg/ml) and 30 nL of AAV2/1-TRE-Cre into the eye through a pulled glass micropipette using the Nanoject. For simultaneous recording of pre- and postsynaptic activity in the SC, P0 mouse pups were injected with an AAV cocktail consisting of 400 nL of AAV2/2-pCAG-FLEX-GFP and 30 nL of AAV2/1-TRE-Cre into the eye through a pulled glass micropipette prior to AAV injections into the SC at P1 (see below). For wide-field single-photon calcium imaging of RGC axons in bulk, we injected 400 nL of AAV1-Syn-GCaMP6s (#100843-AAV1, Addgene) (titer ≥ 1 × 10^13^ vg/ml) into both eyes through a pulled glass micropipette using the Nanoject.

Superior colliculus injections

AAV injections into the SC was performed as previously described (*10*). Briefly, P1 mice were anesthetized using hypothermia and were maintained on a cold metal plate for the duration of the procedure. A small cut was made in the skin above a hemisphere of the SC. The pipette attached to the Nanoject was gently lowered to 200 μm below the surface of the skull. 200 nL of AAV2/9-Syn-NES-jRGECO1a (#100854-AAV9, Addgene) (titer ≥ 3 × 10^13^ vg/ml) was injected at a rate of 2 nL/sec. The pipette was slowly retrieved 1 min after the injection. The pups were allowed to recover for 20 min on a heating pad before being returned to their mothers.

Surgical procedure for in vivo imaging

Mice were surgically implanted with a cranial window as previously described (*10, 21*). Briefly, P8-P9 mice were anesthetized with isoflurane at 2.5% and placed on a heating pad set to 37 °C (HTP-1500, Adroit). After removal of the skin and fascia layers above the skull, steel head posts were secured to the exposed skull using cyanoacrylate (Maxi-Cure, Bob Smith Industries). Isoflurane was then adjusted to 1.5% as necessary to maintain a stable respiratory rate. An approximately 3 mm oval craniotomy was performed above the SC and the dura was carefully removed. A glass coverslip was secured using cyanoacrylate to cover the cranial window. After the surgery, mice were allowed to recover for 90 min on the heating pad with oxygen delivered before imaging sessions. Correlated spontaneous activities did not occur in deeply anesthetized mice and emerged about 30 min after recovery from isoflurane anesthesia (*21*).

In vivo two-photon imaging

We used a movable objective two-photon microscope (MOM) with a Janelia wide-path design and galvo-resonant scanner (Sutter Instruments), as previously described (*53*). Excitation light was provided by a Ti:Sapphire laser (MaiTai eHP DeepSee, Spectra-Physics) with built-in dispersion compensation. Laser intensity into the microscope was controlled using a Pockels cell (Conoptics). The laser was focused on the brain using an objective with a 1.7 mm WD and 1.0 NA (Plan-Apochromat 20×, Zeiss). Fluorescence emissions were reflected into the collection path by a FF735Di-02 dichroic mirror (Semrock), filtered with an ET500lp longpass filter (Chroma) and then split by a T565lpxr dichroic mirror (Chroma) into two GaAsP PMTs (H10770PA-40, Hamamatsu) with ET525/50m-2p (Chroma) and ET605/70m-2p (Chroma) filters for detection of green and red photons, respectively. The two-photon microscope was controlled using ScanImage (Vidrio Technologies).

For in vivo two-photon imaging of both axon dynamics and firing of a single axon arbor, we selected contralaterally projecting RGC axons expressing GFP strongly enough to capture its whole axon arbor in the SC. Typically, 1-3 separated axons were well-labeled by GFP in a hemisphere of the SC at P8-P9. We selected GFP-positive axons that formed dense terminal zones within 200 μm from the surface of the SC. Expression of GCaMP6s in GFP-positive axons was confirmed by two-photon imaging for GCaMP6s with illumination at 1,010-1,020 nm prior to imaging sessions. When several axons were labeled in the field of view, we distinguished a single RGC axon arbor from the others by the different timing of individual axon firing. For acquiring z-stacks of whole axon arbors, GFP was excited at 920 nm. Z-stack images with 405 × 405 μm or 270 × 270 μm field of views were acquired at 2 μm intervals within a depth of 200 μm below the surface of the SC (in the stratum griseum superficiale (SGS)). Total laser power delivered to excite GFP up to a 200 μm depth was <95 mW, within previously described limits for thermal damage (*53, 54*). To reduce motion artifacts during z-stack acquisitions, mice were shortly anesthetized with isoflurane at 1.5%. Each z-stack was captured within 4 min, allowing spontaneous retinal waves to recover immediately after stopping the isoflurane exposure. To block NMDA-R activity, 15 μl of 1mM MK-801 (#0924, Tocris) was intraperitoneally injected into the mice 30 min prior to imaging sessions. Controls for MK-801 were non-injected animals. For chronic treatments of MK-801, mice were intraperitoneally injected with 15 μl of 1mM MK-801 or saline (control) every 24 hr from P5 to P8. To exclude a possibility that GFP-expressing RGC axons in Ai162; β2^fl/-^ did not express GCaMP6s, we only used GFP-expressing axons with axon firing occurring at least once before the imaging session.

For in vivo two-photon dual-color calcium imaging, GCaMP6s and jRGECO1a were simultaneously excited at 1,010-1,020 nm, with total laser power <55 mW. At this laser power, GFP was barely excited at 1,010-1,020 nm, minimizing GFP photobleaching during the 90-min calcium imaging session. A 405 × 405 μm field of view was imaged at a depth around 40-70 μm below the surface of the SC. Images were acquired at a resolution of 512 × 512 pixels (16-bit pixel depth) at 15.25 Hz using a galvo-resonant scanner and were subsequently averaged to produce a final framerate of 5.08 Hz. Imaging was performed on unanesthetized, head-fixed mice in a dark box. During the recordings, mouse pups were placed on a heating pad and were loosely surrounded by cotton gauze.

For in vivo two-photon glutamate imaging, iGluSnFR3 was excited at 1,000 nm, with total laser power <55 mW. Because iGluSnFR3 signals were much weaker than GCaMP6s and GFP signals, contamination of GFP signals into iGluSnFR3 signals was significant even though GFP signals were scarce at 1,000 nm. Therefore, we used tdTomato to label single axons instead of GFP. Because of rapid photobleaching of tdTomato during glutamate imaging, we performed glutamate imaging after taking z-stacks of axon arbors labeled by tdTomato. A 202.5 × 202.5 μm field of view was imaged at a depth around 40-70 μm below the surface of the SC. Images were acquired at a resolution of 512 × 512 pixels (16-bit pixel depth) at 15.25 Hz using a galvo-resonant scanner and were subsequently averaged to produce a final framerate of 7.625 Hz. Movies for iGluSnFR3 were continuously acquired for 6 min in each recording session. For acquiring optical section z-stacks of whole axon arbors, tdTomato was excited at 1,000 nm. Z-stack images with 270 × 270 μm fields of view were acquired at 2 μm intervals within a depth of 200 μm below the surface of the SC.

For in vivo two-photon multiplane calcium imaging, an electro-tunable lens (EL-16-40-TC-VIS-5D-M26, Optotune AG) was placed close to the rear stop of the objective for quick tuning of the focal plane. GCaMP6s was excited at 920 nm at the same laser power across multiple planes. A 405 × 405 μm field of view was imaged at three optical planes 20, 70 and 120 μm below the surface of the SC. Images were acquired at a resolution of 512 × 512 pixels (16-bit pixel depth) at 3.72 Hz per optical plane using a galvo-resonant scanner. Imaging was performed on unanesthetized, head-fixed mice in a dark box. Movies were continuously acquired for 15 min in each recording session.

Wide-field single-photon calcium imaging

Wide-field calcium imaging was performed as previously described (*10*). Briefly, images were collected at 10 Hz using a sCMOS camera (pco.edge 4.2, PCO) coupled to a Zeiss AxioZoom v.16 stereo zoom microscope with a PlanNeoFluar Z 1×/0.25 objective at 56.3× zoom. Illumination was provided by an LED source (X-Cite XLED1) with blue light (470 nm, Chroma ET470/20x) for GCaMP6s. Fluorescence emissions were filtered using a dichroic (Chroma T495lpxr) and emission filter (Chroma ET525/50m). Imaging was performed on unanesthetized, head-fixed mice in a dark box after a 90-min recovery from isoflurane anesthesia and the surgical procedure. Movies were continuously acquired for 15 min in each recording session. During the recordings, mouse pups were placed on a heating pad and were loosely surrounded by cotton gauze.

Calcium image preprocessing

Calcium imaging movies were preprocessed as previously reported(*10, 53*), with modifications specified below. For preprocessing of two-photon calcium imaging data, raw two-photon imaging movies were corrected for motion artifacts using Suite2p package (*54*). Motion-corrected frames were then smoothed with a Gaussian filter (σ = 2). Using custom routines written in MATLAB, slow drifts in the baseline fluorescence of each pixel were removed using top hat filtering across time (50 frame filter object) with the built-in MATLAB function “imtophat”, and the ΔF/F = (F_t_-F_0_)/F_0_ was calculated for each pixel using the 10th percentile value for each pixel across time as F_0_. For preprocessing of wide-field calcium imaging data, raw movies were rotated to align the anterior-posterior axis with the vertical direction. Using custom routines written in MATLAB, fluorescence traces were top-hat filtered to remove bleaching effects, and the ΔF/F was calculated for each pixel using the 10th percentile value for each pixel across time as F_0_.

Calcium signal detection

For in vivo two-photon calcium imaging data, ROI extraction and calcium event detection were performed by custom routines written in MATLAB. We identified all branch terminal positions for individual axons and calculated the center of each axon arbor from all its branch terminals. We then defined branch terminal positions to be distal and central if their distances to the center of its axon arbor were more than the 95th percentile of the distances and less than the 5th percentile, respectively. We defined distal and central ROIs as the regions within a circle of 7.9 μm radius from the distal and central branch terminals, respectively. We then used the average fluorescence intensity value across the pixels belonging to the ROI to define the calcium signals at each time point and obtained calcium traces. A calcium event was identified if the calcium signals were above the threshold (3 std + mean) and had a duration of 3 frames (0.59 sec) or more. Single-axon firing and retinal waves measured in the ROI were considered as synchronous if the calcium events from the respective calcium traces overlapped for 3 frames (0.59 sec) or more. Frequencies of calcium events were determined by dividing the number of calcium events by the length of time for each recording. A 30-min movie was used for calculating the frequency of calcium events, and a 45-min movie was used for calculating the fraction of synchronization between single-axon firing and retinal waves at the distal and central regions of the axon arbor. Fraction of synchronized firing were determined by dividing the number of events with “synchronized firing between axon firing and retinal waves” by the total number of events with “synchronized firing between axon firing and retinal waves”, “single axon firing without retinal waves”, and “retinal waves without single axon firing”. We calculated the frequency of calcium events and the fraction of synchronization for all distal branch terminals and central branch terminals. Different distal branch terminals of the same arbor could exhibit different synchronized firing with retinal waves. We then quantified the mean of these values across distal or central branch terminals to define the value for a single axon. Frames with excessive motion artifacts which were visible on “View registered binary” in the Suite2p GUI were not included for analyses. All animals with appropriate expression of GCaMP6s and jRGECO1a to perform automated calcium event analysis were included for analyses. For wide-field calcium imaging data, GCaMP6s signals were measured in ROIs of a 20.5 μm radius.

Glutamate image preprocessing

For preprocessing of two-photon glutamate imaging data, raw movies were corrected for motion artifacts using Suite2p package (*54*). Motion-corrected frames were then smoothed with a Gaussian filter (σ = 1). Using custom routines written in MATLAB, slow drifts in the baseline fluorescence of each pixel were removed using top-hat filtering across time (50 frame filter object) with the built-in MATLAB function “imtophat”, and the ΔF/F = (F_t_-F_0_)/F_0_ was calculated for each pixel using the 10th percentile value for each pixel across time as F_0_.

Glutamate image analysis

Max ΔF/F maps for iGluSnFR3 were generated using the ImageJ “z project” function from preprocessed movies. Glutamate release sites (GRSs) were defined as axon segments with significant signals (ΔF/F above mean + 2 std of the max ΔF/F map) and containing at least 6 pixels (1 μm^2^). Such GRSs were automatically identified using the ImageJ function “analyzed particles” from max ΔF/F maps. GRSs not overlapped with axon segments were excluded from this analysis. We defined 'branch points with presynaptic sites' as branch points within 2.5 μm distance from a GRS. Max ΔF/F in each GRS was calculated with the ImageJ “ROI manager” function. For branch point analysis, we only analyzed branch points in the optical plane where glutamate imaging of single RGC axons was performed. We defined “persisting branch point” as branch points that were persistent for 2 hr, “eliminated branch point” as branch points that existed at 0 hr but were eliminated at 2 hr, and “added branch point” as branch points that did not exist at 0hr but appeared at 2hr. Frames with excessive movement artifacts which were visible on “View registered binary” in the Suite2p GUI were not included for analyses.

Generation of seed-based correlation maps

Seed-based correlation analysis was performed by custom routines written in MATLAB. To generate correlation maps between individual axon firing and retinal waves, GCaMP6s signals from each axon arbor served as correlation seeds and were used to compute correlation coefficient values between the timecourses of the seed signals and jRGECO1a signals at each pixel of the images with the built-in MATLAB function “corr”. Frames with excessive motion artifacts which were visualized on “View registered binary” in the Suite2p GUI were not included for calculating correlation coefficient values. Movies shorter than 60 minutes after removing frames with excessive movement artifacts were not included for this analysis.

For wide-field calcium imaging data, GCaMP6s signals in randomly selected ROIs of a 20.5 μm radius in the SC served as correlation seeds and were used to compute correlation coefficient values with every pixel in the images with built-in MATLAB function “corr”. ROIs in the midline region were selected within 200 μm from the midline, and ROIs in the lateral region were selected at least 400 μm away from the midline. A 15-min movie was used for wide-field calcium imaging data. For comparison between seed-based correlation maps, the contour of the highly correlated area where correlation coefficient values were above the threshold (4 std + mean) was drawn with the built-in MATLAB function “contour”. Then, the width and height of the contoured area were measured and used for calculating the ratio of the length along the mediolateral axis to the length along the rostrocaudal axis. Orientation axes of the contoured area were measured with the built-in MATLAB function “regionprops”. Four ROIs in the lateral region and two ROIs in the medial region were randomly selected per animal to calculate the mean length ratios and orientation of the axes.

Axon branch dynamics analysis

All two-photon image stacks of RGC axon arbors were denoised using CANDLE non-local means denoising software implemented in MATLAB (*55*). When motion correction was required, z-stacks were aligned using Linear Stack Alignment with SIFT plugin in Fiji/ImageJ. Single axon tracing and axon branch terminal plotting were performed using the segmentation tool of 3D tracing in the Simple Neurite Tracer (SNT) plugin in Fiji/ImageJ (*56*). Axon branch dynamics were analyzed by comparing the same axon at 0 hr and 2 hr time points using the SNT. We only analyzed branches that measured at least 5 μm in length to exclude axonal boutons and filopodia. We defined elongated and retracted branches as branches that were persistent for 2hr but their lengths at 2hr were 3 μm longer and shorted than that at 0 hr, respectively. Correlation coefficient values in seed-based correlation maps were extracted at 0 hr positions of stable, eliminated, elongated and retracted axon branch terminals and at branch points of added branches using custom routines written in MATLAB. After extracting the correlation coefficient for individual branches, means of correlation coefficient were calculated for each branch type per axon. For this analysis, we included all axon branches within 40 μm from the optical plane at which dual-color calcium imaging was performed for generating the correlation map because retinal waves were highly synchronized within 40 μm from the optical plane (fig. S1H). Distances from the center of a single axon arbor to stable and eliminated branch terminals and added branch points were normalized by the mean distances to the axon arbor center of all branch terminals. The mean of normalized values for a given type of axon branches would be one if axon branch positions were randomly distributed and would be smaller than one if axon branch positons were distributed near the center. For z-score analysis, distances of individual branches from the center of its axon or correlation coefficient of individual branches were converted to z-score with the built-in MATLAB function “zscore”, then, the mean of z-score was calculated for each branch type per axon.

Covariance error ellipse

Ellipse fitting for covariance error of axon terminal positions was performed by custom routines written in MATLAB. Eigenvalues and eigenvectors were calculated from the 2D projection of all axon terminal positions in a single RGC axon by the principal component analysis (PCA). Eigenvectors represented the directions of the major and minor axis of the ellipse. The lengths of major and minor axis of the ellipse were calculated as:

$$\begin{matrix} eigenvalue_{1}>eigenvalue_{2} \\ Major axis length=2\sqrt{s\times eigenvalue_{1}} \\ Minor axis length=2\sqrt{s\times eigenvalue_{2}} \end{matrix}$$

where *s* is a value of chi-square probability with a 90% confidence interval for 2 degrees of freedom. Orientation angles of ellipses were calculated from absolute XY values of the eigenvectors for the major axis with the built-in MATLAB function “atan2”. The angles were in the range of 0°-90°. For FRMD7 and related control experiments, we only used RGC axons in the lateral region of the SC.

Statistical analysis

Data sets were analyzed and plotted using custom routines implemented in MATLAB or in GraphPad Prism 9. Data were analyzed by Wilcoxon rank sum test, paired *t* test, Wilcoxon signed rank test or one-way ANOVA with Dunnett’s multiple comparisons test. Additional details on sample sizes, statistical test, significant levels for each experiment can be found in figure legends and Results. No statistical analysis was used to pre-determine sample sizes. For all the box plots, the central line indicates the median, the bottom and top edges indicate the 25th and 75th percentiles of the data. Stars in figures indicate the following: * p<0.05, ** p<0.01, *** p<0.001.

Code availability

Custom MATLAB routines used in this study can be found at: https://github.com/Nao-Ma/Crair_lab.


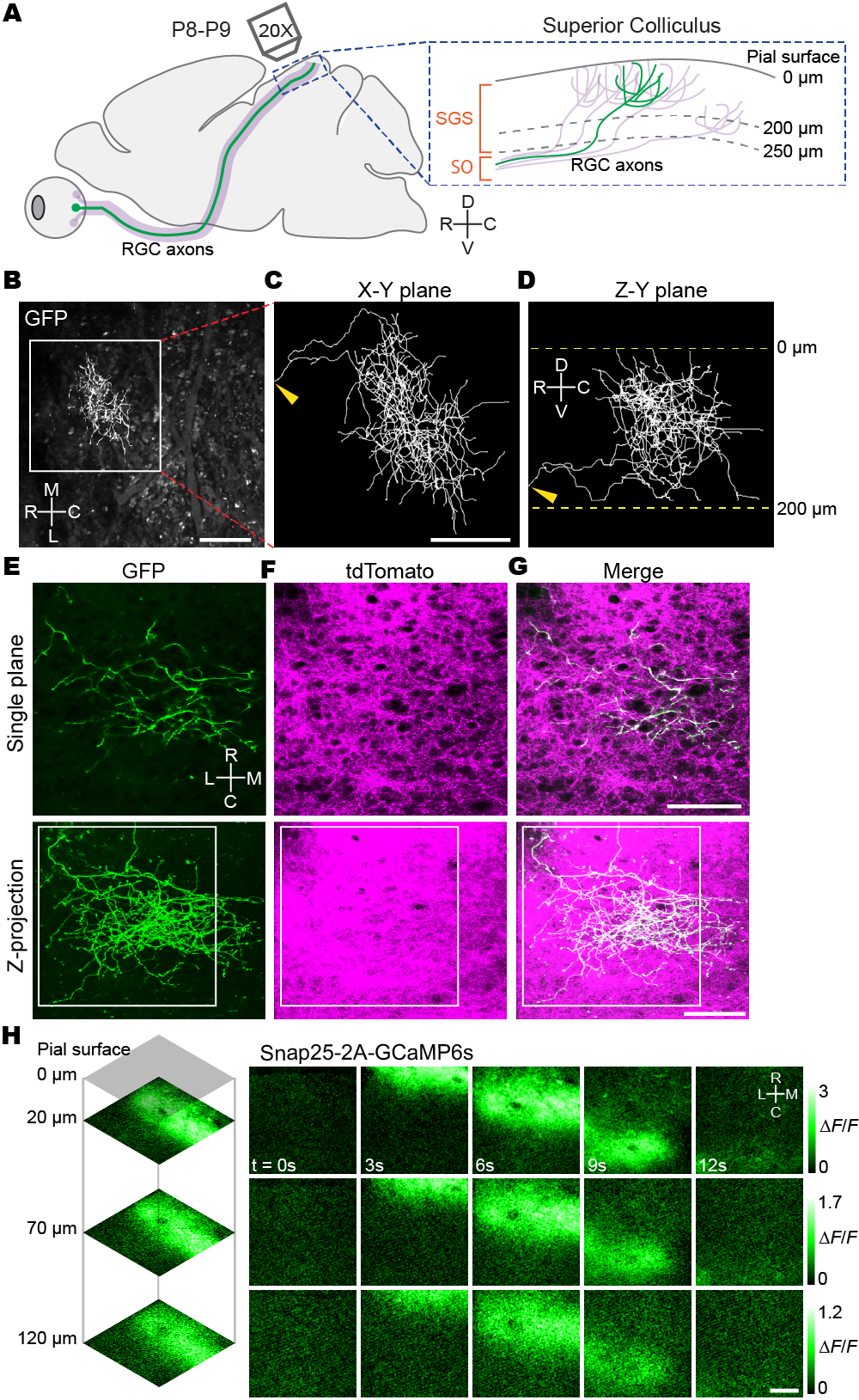


Fig. S1. Single RGC axons and retinal waves observed across depths in the SC in vivo.

(**A**) Schematic illustration of the retinocollicular projection in a parasagittal mouse brain. Directions R, C, D, and V correspond to rostral, caudal, dorsal, and ventral in the brain unless otherwise stated. Additional abbreviations: SGS, stratum griseum superficiale; SO, stratum opticum. (**B**) In vivo two-photon imaging of a single RGC axon in the SC at P8. Directions R, L, M and C correspond to rostral, lateral, medial and caudal in the SC unless otherwise stated. The boxed area is magnified in (C). Scale bar, 100 μm. (**C** and **D**) Projection of traced axon branches within a depth of 200 μm below the surface of the SC. X-Y (C) or Z-Y (D) planes of reconstructed branches are shown. Arrow heads indicate the shaft of the primary axon. Yellow dotted line indicates depths at 0 μm and 200 μm from the pial surface of the SC, respectively. Scale bar, 100 μm. (**E**-**G**) A single plane (top) and z-projection (bottom) of a single RGC axon (E) and bulk-label RGC axons (F) in the SC from in vivo two-photon imaging at P8. Sparse GFP expression in RGCs was achieved by intravitreal injections of AAV2/1-TRE-Cre, AAV2/2-CAG-FLEX-EGFP and AAV2/2-CAG-tdTomato in Ai162 mice. The single RGC axon and neighboring RGC axons were highly intertwined. The boxed area in bottom panels is magnified in top panels. Scale bars, 100 μm. (**H**) In vivo two-photon multi-plane calcium imaging of retinal waves in the SC of Snap25-2A-GCaMP6s mouse at P8. Three-plane imaging at 50-μm intervals are shown. The same laser power was used to excite GCaMP6s for each plane, and therefore, signals decreased with increasing depth. Retinal waves were highly synchronized along z-axis within the upper SGS. Based on this result, we performed correlation analyses for all axon branches within 40 μm from the optical plane of dual-color calcium imaging which was at a depth around 40-70 μm below the surface of the SC (see Materials and Methods). Scale bar, 100 μm.


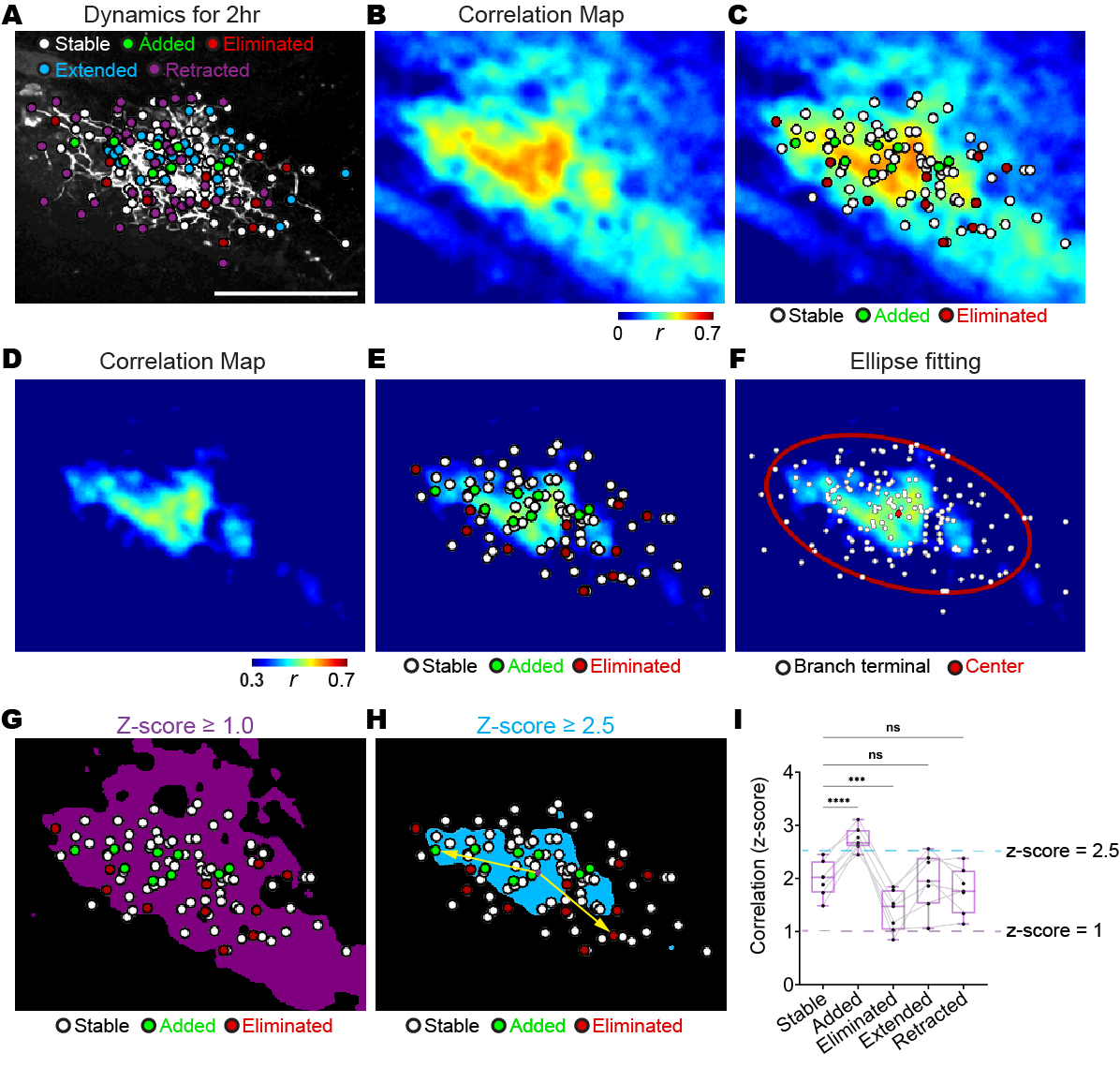


Fig. S2. Added and eliminated branches are preferentially located in the regions where axon firing was relatively synchronized and asynchronized with retinal waves, respectively.

(**A**-**C**) The same panels as Fig. 1, L to N. (**D**) The same data as (A) but only showing area in the correlation map with *r* (Pearson’s correlation coefficient) above 0.3. (**E**) Stable and eliminated branch terminals and positions where newly added branches emerged from were plotted on the correlation map of (D). Most of the newly added branches resided in the area with *r* > 0.3, whereas eliminated branch terminals were outside this area. (**F**) Axon branch terminals (white) and their covariance ellipse with a 90% confidence interval (red) were plotted over the correlation map. The shape of correlation coefficient map did not match the shape of the covariance ellipse. The axon firing was more correlated with retinal waves in the left half of the covariance ellipse. (**G**) The area with correlation coefficient more than 1 std + mean was labeled by magenta. Most of branches were located within the area. (**H**) Area with correlation coefficient more than 2.5 std + mean was labeled by cyan. Most of added branches were located within the area. This area is not similar with the terminal zone of the axon arbor. Arrows indicate representative added and eliminated branches that were distributed at similar distances to the center of the axon arbor along the long axis of the axon innervation area but had very different correlation coefficients. (**I**) Means of z-score (stable, 2.0 ± 0.1; added, 2.7 ± 0.1, p < 0.0001; eliminated, 1.4 ± 0.1, p = 0.0001; extended, 01.9 ± 0.2, p = 0.991; retracted, 1.8 ± 0.2, p = 0.23, one-way ANOVA with Dunnett’s multiple comparison test, n = 7 axons from 7 animals). ***p < 0.001, ****p < 0.0001. Data are mean ± SEM.


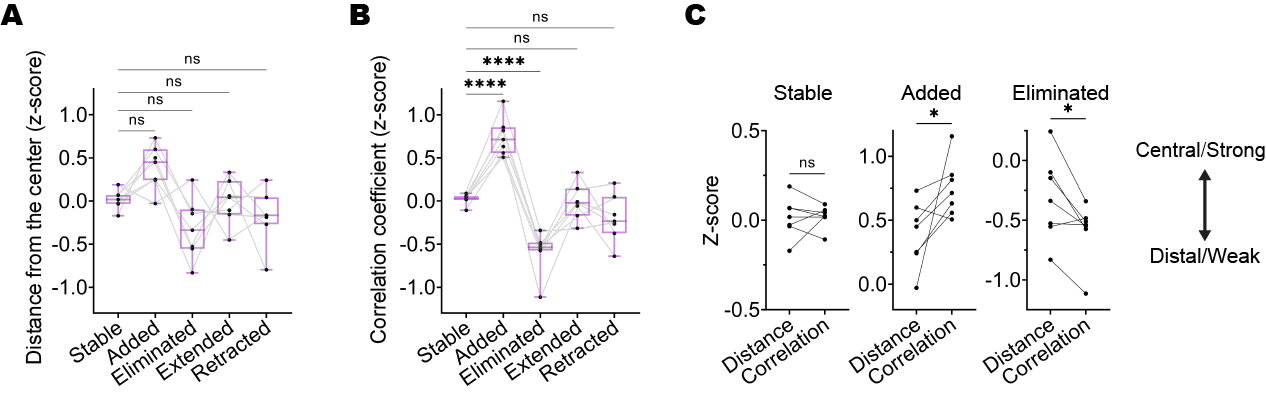


Fig. S3. Positions of added and eliminated axon branches are more related to the correlation between axon firing and retinal waves than the distance from the center of the axon.

(**A**) Distances of individual branches from the center of its axon were converted to z-score, and the means of sign-inverted z-score were calculated for each branch type per axon (stable, 0.02 ± 0.04; added, 0.39 ± 0.09, p = 0.06; eliminated, -0.32 ± 0.12, p = 0.11; extended, 0.01 ± 0.09, p = 1.00; retracted, -0.19 ± 0.11, p = 0.48, one-way ANOVA with Dunnett’s multiple comparison test). (**B**) Correlation coefficients of axon firing with retinal waves at individual branches were converted to z-score, and the means of z-score were calculated for each branch type per axon (stable, 0.02 ± 0.02; added, 0.75 ± 0.08, p < 0.0001; eliminated, -0.59 ± 0.09, p < 0.0001; extended, -0.01 ± 0.07, p = 0.99; retracted, -0.20 ± 0.10, p = 0.17, one-way ANOVA with Dunnett’s multiple comparison test). (**C**) Comparison of z-score distribution between the distance from axon centers and correlation coefficient. Data points from the same axon were paired (stable, p = 0.41; added, p = 0.016; eliminated, p = 0.023; one-tailed Wilcoxon signed-rank test). All the z-scores for distances have their signs inverted. n = 7 axons from 7 animals. *p < 0.05, ****p < 0.0001. Data are mean ± SEM.


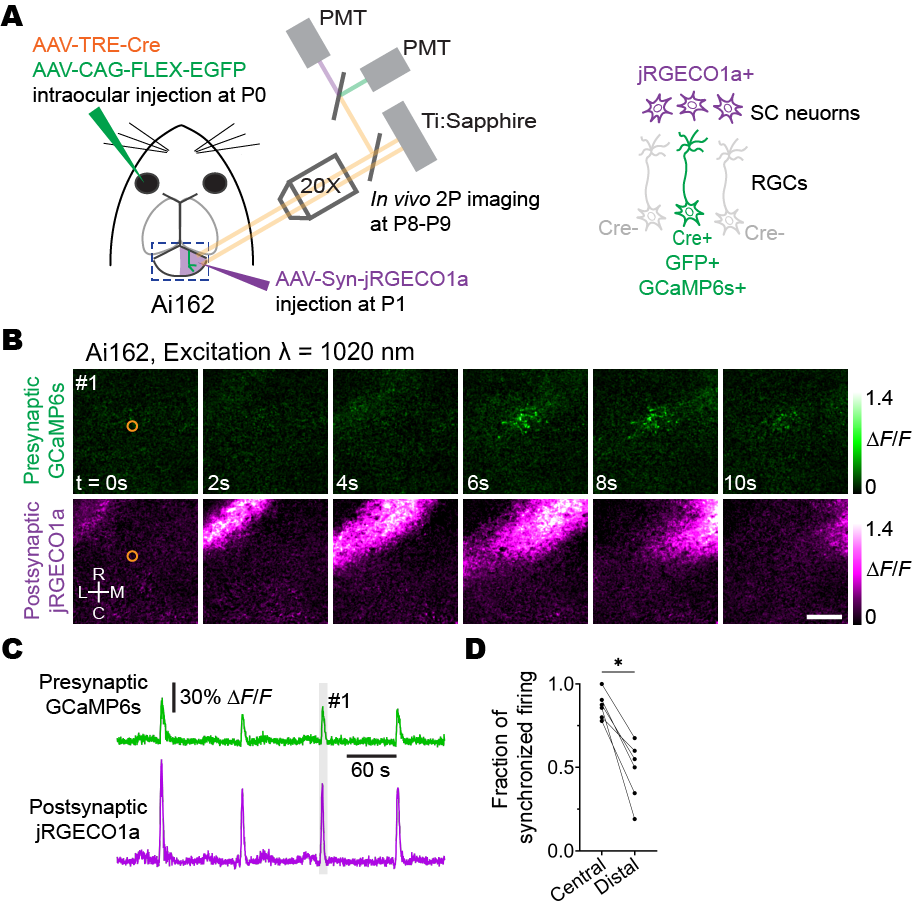


Fig. S4. Addition and elimination of axon branches is related to synchrony of axon branch firing with postsynaptic waves.

(**A**) Schematic of the experimental approach for simultaneous in vivo two-photon imaging of single RGC axon branch dynamics and dual-color calcium imaging of axonal activity and postsynaptic waves in the SC. Stochastic expression of Cre in a few RGCs was achieved by intravitreal injections of AAV2/1-TRE-Cre in Ai162 mice which harbor CAG-LSL-tTA2 and TRE-LSL-GCaMP6s alleles, and expression of jRGECO1a in neurons of the SC was achieved by injection of AAV2/9-Syn-NES-jRGECO1a into the SC one day after eye injection. After the AAV injections, only a few RGC axons expressed GFP and GCaMP6, and most of SC neurons expressed jRGECO1a. (**B**) ΔF/F montages of single-axon firing (GCaMP6s) and of a postsynaptic wave (jRGECO1a). Orange circles correspond to a central ROI for traces in (C). Directions R, L, M and C correspond to rostral, lateral, medial and caudal in the SC unless otherwise stated. Scale bar, 100 μm. (**C**) Traces (ΔF/F) of presynaptic single-axon firing (GCaMP6s) and postsynaptic waves (jRGECO1a) were measured in the ROI indicated in (B). Gray area (#1) indicates the period depicted in montages (B). (**D**) Fraction of synchronization between single-axon firing and postsynaptic waves at central and distal regions of the axon arbor. Data points from the same axon were paired (central, 0.87 ± 0.03; distal, 0.48 ± 0.07; p = 0.02, one-tailed Wilcoxon signed-rank test, n = 6 axons from 6 animals at P8-P9). *p < 0.05.


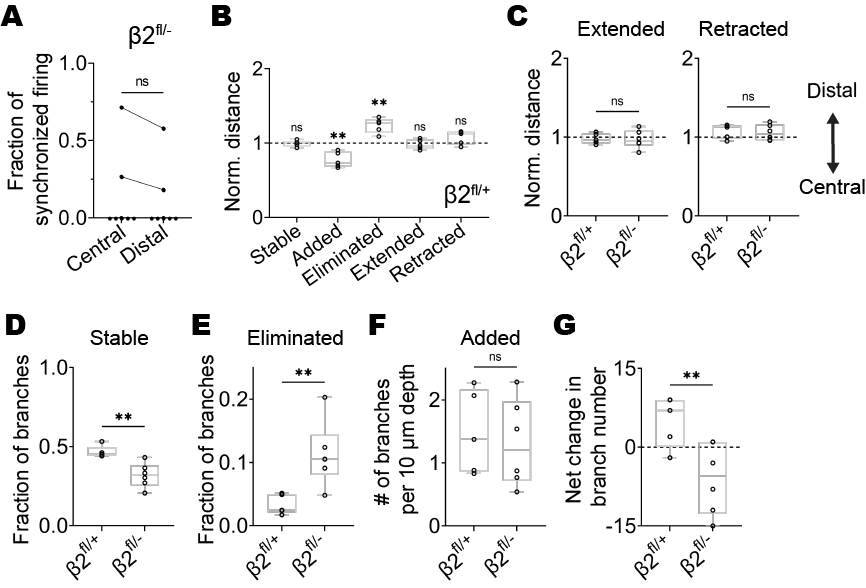


Fig. S5. Axon branch dynamics in single-cell β2-nAChR-knockout RGCs.

(**A**) β2-nAChR was knocked out in only a few RGCs by intravitreal injections of AAV2/1-TRE-Cre in Ai162; β2^fl/-^ mice. The fraction of synchronization between single-axon firing and retinal waves at central regions of the axons was comparable with that at distal regions in β2-nAChR knockout axons. Data points from the same axon were paired (central, 0.14 ± 0.25; distal, 0.11 ± 0.05; p = 0.20, one-tailed Wilcoxon signed-rank test, n = 7 axons from 7 animals). (**B**-**G**) AAV2/1-TRE-Cre, AAV2/2-CAG-FLEX-EGFP and AAV2/1-Syn-jRGECO1a were injected into an eye of Ai162; β2^fl/+^ or Ai162; β2^fl/-^ mice at P0, and z stacks of single RGC axon arbors were acquired at a 2-hr interval at P8. (B) Normalized distances from the center of single axons to their stable, eliminated, extended and retracted branch terminals and added branch points in Ai162; β2^fl/+^ mice. Distances were normalized by the mean distance from the center to each branch. The normalized values would be one if axon branch positions were randomly distributed and would be smaller than one if axon branch positions were distributed near the center (stable: p = 0.84; added: p = 0.008; eliminated: p = 0.006; extended: p = 0.58; retracted: p = 0.14; one sample t-test, hypothetical mean = 1.0). (C) Normalized distances from the center of single axon arbors to their extended and retracted branch terminals. Extended: β2^fl/+^, 0.98 ± 0.03; β2^fl/-^, 0.97 ± 0.04; p = 0.47, one-tailed Wilcoxon rank sum test. Retracted: β2^fl/+^, 1.08 ± 0.04; β2^fl/-^, 1.06 ± 0.04; p = 0.47, one-tailed Wilcoxon rank sum test. (D) Fraction of stable branches (β2^fl/+^, 0.47 ± 0.02; β2^fl/-^, 0.32 ± 0.02; p = 0.002, one-tailed Wilcoxon rank sum test). (E) Fraction of eliminated branches (β2^fl/+^, 0.033 ± 0.006; β2^fl/-^, 0.113 ± 0.019; p = 0.009, one-tailed Wilcoxon rank sum test). (F) Number of added branches per 10 μm depth (β2^fl/+^, 1.5 ± 0.3; β2^fl/-^, 1.3 ± 0.3; p = 0.33, one-tailed Wilcoxon rank sum test). (G) Net change in branch number (β2^fl/+^, 5.0 ± 1.9; β2^fl/-^, -6 ± 2.5; p = 0.009, one-tailed Wilcoxon rank sum test). (B-G) β2^fl/+^, n = 5 axons from 5 animals at P8; β2^fl/-^, n = 6 axons from 6 animals at P8. For all the box plots, the central line indicates the median, the bottom and top edges indicate the 25th and 75th percentiles of the data. *p < 0.05, **p < 0.01. Data are mean ± SEM.


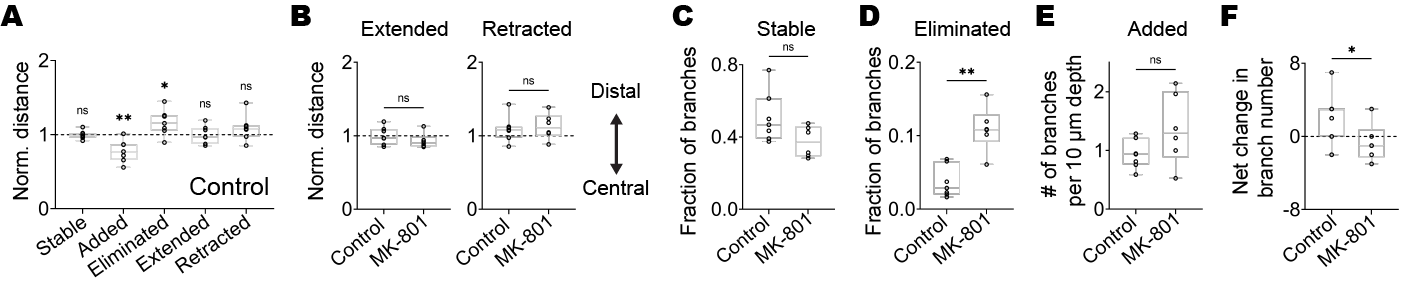


Fig. S6. Single RGC axon branch dynamics after MK-801 treatment.

AAV2/1-TRE-Cre, AAV2/2-CAG-FLEX-EGFP and AAV2/1-Syn-jRGECO1a were injected into the eye of Ai162 mice at P0-P1. MK-801 was intraperitoneally injected into mice 30 min prior to imaging sessions. Z stacks of single RGC axon arbors were acquired at a 2-hr interval at P8-P9. (**A**) Normalized distance from the center of single axon arbors to their stable, eliminated, extended and retracted branch terminals and added branch points in control mice. Distance was normalized by the mean distance from the center to each branch. The normalized value would be one if axon branch positions were randomly distributed and would be smaller than one if axon branch positions were distributed near the center (stable: p = 0.94; added: p = 0.008; eliminated: p = 0.049; extended: p = 0.98; retracted: p = 0.22; one sample t-test, hypothetical mean = 1.0). (**B**) Normalized distance from the center of single axon arbors to their extended and retracted branch terminals. Extended: control, 1.00 ± 0.04; MK-801, 0.93 ± 0.04; p = 0.15, one-tailed Wilcoxon rank sum test. Retracted: control, 1.09 ± 0.06; MK-801, 1.13 ± 0.07; p = 0.42, one-tailed Wilcoxon rank sum test. (**C**) Fraction of stable branches (control, 0.51 ± 0.05; MK-801, 0.37 ± 0.03; p = 0.10, one-tailed Wilcoxon rank sum test). (**D**) Fraction of eliminated branches (control, 0.037 ± 0.008; MK-801, 0.109 ± 0.011; p = 0.005, one-tailed Wilcoxon rank sum test). (**E**) Number of added branches per 10 μm depth (control, 0.93 ± 0.09; MK-801, 1.4 ± 0.2; p = 0.18, one-tailed Wilcoxon rank sum test). (**F**) Net change in branch number (control, 2.3 ± 1.0; MK-801, -0.7 ± 0.8; p = 0.04, one-tailed Wilcoxon rank sum test). (A-F) Control, n = 7 axons from 7 animals at P8-P9; MK-801, n = 6 axons from 6 animals at P8-P9. For all the box plots, the central line indicates the median, the bottom and top edges indicate the 25th and 75th percentiles of the data. *p < 0.05, **p < 0.01. Data are means ± SEM.


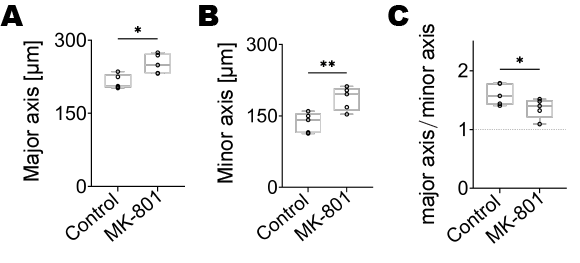


Fig. S7. Chronic blockade of NMDARs disrupts mediolateral orientation of RGC axon arbors in the lateral SC.

MK-801 or saline (control) was intraperitoneally injected into mice every 24 hr from P5 to P8. Z stacks of single RGC axon arbors were acquired at P8. (**A**) Lengths of the major axes of covariance ellipses with a 90% confidence interval calculated from axon branch terminal positions of individual axon arbors (control, 214.5 ± 6.0 μm; MK-801, 251.5 ± 8.0 μm; p = 0.016, one-tailed Wilcoxon rank sum test). (**B**) Lengths of minor axes of covariance ellipse (control, 136.0 ± 8.3 μm; MK-801, 186.8 ± 9.9 μm; p = 0.008, one-tailed Wilcoxon rank sum test). (**C**) The ratio of the length of the major axis over the length of the minor axis of the covariance ellipse (control, 1.60 ± 0.07; MK-801, 1.36 ± 0.07; p = 0.048, one-tailed Wilcoxon rank sum test). (A-C) All axons were from lateral regions. Control, n = 5 axons at P8; MK-801, n = 5 axons at P8. *p < 0.05, **p < 0.01. Data are means ± SEM.


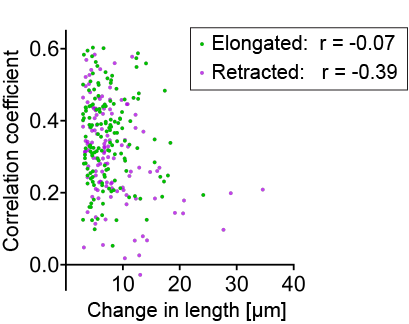


Fig. S8. Scatter plot of the correlation coefficient and the change in length during the imaging period for elongated and retracted axon branches.

Correlation coefficient of signal axon firing and retinal waves for elongated axon branches (green) and retracted axon branches (magenta) is plotted against the change in branch lengths during the 2hr imaging period (Elongated: r = -0.07, p = 0.19, n = 153 branches; Retracted: r = -0.39, p < 0.0001, n = 127 branches, Pearson’s correlation, N = 7 axons from 7 animals at P8-P9).

**Movie S1.**

A single RGC axon shown in (Fig. 1B) was 3D reconstructed within a depth of 200 μm below the surface of the SC at P8.

**Movie S2.**

In vivo two-photon dual-color calcium imaging of the retinal ganglion cell (RGC) axon firing and retinal waves in the superior colliculus (SC) at P8. Orange circles correspond to the central ROI for traces in (Fig. 1F). Movies were recorded at 5.08 Hz and played at 25.4 frames/second.

**Movie S3.**

In vivo two-photon multi-plane calcium imaging of the retinal waves in the superior colliculus (SC) at P8. Movies were recorded with 50 μm steps at 3.72 Hz per individual plane and played at 37.2 frames/second.

**Movie S4.**

A single axon shown in (Fig. 1J) was 3D reconstructed within 40 μm from the optical plane at which dual-color calcium imaging was performed for generating the correlation map. Behaviors of individual branches over the 2 hr interval were colorized.

**Movie S5.**

In vivo two-photon dual-color calcium imaging of single RGC axon firing and postsynaptic waves in the SC at P8. Orange circles correspond to the central ROI for traces in (fig. S4C). Movies were recorded at 5.08 Hz and played at 25.4 frames/second.

**Movie S6.**

In vivo two-photon dual-color calcium imaging of single-cell β2-knockout RGC axon firing and retinal waves in the SC of Ai162; β2^fl/-^ at P8. Orange circles correspond to the central ROI for traces in (Fig. 2B). Movies were recorded at 5.08 Hz and played at 25.4 frames/second.

**Movie S7.**

Wide-field single-photon calcium imaging of spontaneous retinal activity in the SC of a wild-type mouse at P9. Movies were recorded at 10 Hz and played at 200 frames/second.

**Movie S8.**

Wide-field single-photon calcium imaging of spontaneous retinal activity in the SC of a FRMD7^tm^ mouse at P9. Movies were recorded at 10 Hz and played at 200 frames/second.

**Movie S9.**

In vivo two-photon dual-color calcium imaging of single RGC axon firing and retinal waves in the SC after MK-801 intraperitoneal injection. Orange circles correspond to the central ROI for traces in (Fig. 5B). Movies were recorded at 5.08 Hz and played at 25.4 frames/second.

**Table S1. (separate file)**

Correlation coefficients and distances of individual branches to their axon centers from all the axons used for (Fig. 1O).

References and Notes

1. L. C. Katz, C. J. Shatz, Synaptic activity and the construction of cortical circuits. *Science* **274**, 1133-1138 (1996).

2. A. D. Huberman, M. B. Feller, B. Chapman, Mechanisms underlying development of visual maps and receptive fields. *Annu Rev Neurosci* **31**, 479-509 (2008).

3. S. Serizawa *et al.*, A neuronal identity code for the odorant receptor-specific and activity-dependent axon sorting. *Cell* **127**, 1057-1069 (2006).

4. X. Nicol *et al.*, cAMP oscillations and retinal activity are permissive for ephrin signaling during the establishment of the retinotopic map. *Nat Neurosci* **10**, 340-347 (2007).

5. M. Cynader, N. Berman, A. Hein, Cats reared in stroboscopic illumination: effects on receptive fields in visual cortex. *Proc Natl Acad Sci U S A* **70**, 1353-1354 (1973).

6. D. H. Hubel, T. N. Wiesel, Binocular interaction in striate cortex of kittens reared with artificial squint. *J Neurophysiol* **28**, 1041-1059 (1965).

7. A. G. Blankenship, M. B. Feller, Mechanisms underlying spontaneous patterned activity in developing neural circuits. *Nat Rev Neurosci* **11**, 18-29 (2010).

8. F. J. Martini, T. Guillamon-Vivancos, V. Moreno-Juan, M. Valdeolmillos, G. Lopez-Bendito, Spontaneous activity in developing thalamic and cortical sensory networks. *Neuron* **109**, 2519-2534 (2021).

9. L. A. Kirkby, G. S. Sack, A. Firl, M. B. Feller, A role for correlated spontaneous activity in the assembly of neural circuits. *Neuron* **80**, 1129-1144 (2013).

10. X. Ge *et al.*, Retinal waves prime visual motion detection by simulating future optic flow. *Science* **373**, (2021).

11. Y. Wang *et al.*, Efferent feedback controls bilateral auditory spontaneous activity. *Nat Commun* **12**, 2449 (2021).

12. H. Mizuno *et al.*, Patchwork-Type Spontaneous Activity in Neonatal Barrel Cortex Layer 4 Transmitted via Thalamocortical Projections. *Cell Rep* **22**, 123-135 (2018).

13. D. O. Hebb, The organization of behavior. *Wiley*, (1949).

14. C. J. Shatz, The developing brain. *Sci Am* **267**, 60-67 (1992).

15. J. Zhang, J. B. Ackman, H. P. Xu, M. C. Crair, Visual map development depends on the temporal pattern of binocular activity in mice. *Nat Neurosci* **15**, 298-307 (2011).

16. S. El-Boustani *et al.*, Locally coordinated synaptic plasticity of visual cortex neurons in vivo. *Science* **360**, 1349-1354 (2018).

17. M. Munz *et al.*, Rapid Hebbian axonal remodeling mediated by visual stimulation. *Science* **344**, 904-909 (2014).

18. M. C. Crair, R. C. Malenka, A critical period for long-term potentiation at thalamocortical synapses. *Nature* **375**, 325-328 (1995).

19. A. Gribizis *et al.*, Visual cortex gains independence from peripheral drive before eye opening. *Neuron* **104**, 711-723 e713 (2019).

20. M. Weliky, L. C. Katz, Correlational structure of spontaneous neuronal activity in the developing lateral geniculate nucleus in vivo. *Science* **285**, 599-604 (1999).

21. J. B. Ackman, T. J. Burbridge, M. C. Crair, Retinal waves coordinate patterned activity throughout the developing visual system. *Nature* **490**, 219-225 (2012).

22. T. A. Seabrook, T. J. Burbridge, M. C. Crair, A. D. Huberman, Architecture, function, and assembly of the mouse visual system. *Annu Rev Neurosci* **40**, 499-538 (2017).

23. T. J. Burbridge *et al.*, Visual circuit development requires patterned activity mediated by retinal acetylcholine receptors. *Neuron* **84**, 1049-1064 (2014).

24. J. Cang, D. A. Feldheim, Developmental mechanisms of topographic map formation and alignment. *Annu Rev Neurosci* **36**, 51-77 (2013).

25. B. K. Stafford, A. Sher, A. M. Litke, D. A. Feldheim, Spatial-temporal patterns of retinal waves underlying activity-dependent refinement of retinofugal projections. *Neuron* **64**, 200-212 (2009).

26. L. Wang *et al.*, Direction-specific disruption of subcortical visual behavior and receptive fields in mice lacking the beta2 subunit of nicotinic acetylcholine receptor. *J Neurosci* **29**, 12909-12918 (2009).

27. J. Cang, L. Wang, M. P. Stryker, D. A. Feldheim, Roles of ephrin-as and structured activity in the development of functional maps in the superior colliculus. *J Neurosci* **28**, 11015-11023 (2008).

28. O. S. Dhande *et al.*, Development of single retinofugal axon arbors in normal and beta2 knock-out mice. *J Neurosci* **31**, 3384-3399 (2011).

29. D. K. Simon, D. D. O'Leary, Development of topographic order in the mammalian retinocollicular projection. *J Neurosci* **12**, 1212-1232 (1992).

30. A. D. Huberman *et al.*, Architecture and activity-mediated refinement of axonal projections from a mosaic of genetically identified retinal ganglion cells. *Neuron* **59**, 425-438 (2008).

31. E. S. Ruthazer, C. J. Akerman, H. T. Cline, Control of axon branch dynamics by correlated activity in vivo. *Science* **301**, 66-70 (2003).

32. J. Y. Hua, M. C. Smear, H. Baier, S. J. Smith, Regulation of axon growth in vivo by activity-based competition. *Nature* **434**, 1022-1026 (2005).

33. H. T. Cline, Activity-dependent plasticity in the visual systems of frogs and fish. *Trends Neurosci* **14**, 104-111 (1991).

34. B. Alsina, T. Vu, S. Cohen-Cory, Visualizing synapse formation in arborizing optic axons in vivo: dynamics and modulation by BDNF. *Nat Neurosci* **4**, 1093-1101 (2001).

35. J. A. Demas, H. Payne, H. T. Cline, Vision drives correlated activity without patterned spontaneous activity in developing Xenopus retina. *Dev Neurobiol* **72**, 537-546 (2012).

36. H. Mizuno *et al.*, NMDAR-regulated dynamics of layer 4 neuronal dendrites during thalamocortical reorganization in neonates. *Neuron* **82**, 365-379 (2014).

37. T. L. Daigle *et al.*, A suite of transgenic driver and reporter mouse lines with enhanced brain-cell-type targeting and functionality. *Cell* **174**, 465-480 e422 (2018).

38. G. J. Broussard *et al.*, In vivo measurement of afferent activity with axon-specific calcium imaging. *Nat Neurosci* **21**, 1272-1280 (2018).

39. M. Meister, R. O. Wong, D. A. Baylor, C. J. Shatz, Synchronous bursts of action potentials in ganglion cells of the developing mammalian retina. *Science* **252**, 939-943 (1991).

40. M. Constantine-Paton, H. T. Cline, E. Debski, Patterned activity, synaptic convergence, and the NMDA receptor in developing visual pathways. *Annu Rev Neurosci* **13**, 129-154 (1990).

41. M. B. Feller, D. P. Wellis, D. Stellwagen, F. S. Werblin, C. J. Shatz, Requirement for cholinergic synaptic transmission in the propagation of spontaneous retinal waves. *Science* **272**, 1182-1187 (1996).

42. A. Bansal *et al.*, Mice lacking specific nicotinic acetylcholine receptor subunits exhibit dramatically altered spontaneous activity patterns and reveal a limited role for retinal waves in forming ON and OFF circuits in the inner retina. *J Neurosci* **20**, 7672-7681 (2000).

43. H. P. Xu *et al.*, An instructive role for patterned spontaneous retinal activity in mouse visual map development. *Neuron* **70**, 1115-1127 (2011).

44. K. Yonehara *et al.*, Congenital nystagmus gene FRMD7 is necessary for establishing a neuronal circuit asymmetry for direction selectivity. *Neuron* **89**, 177-193 (2016).

45. R. D. Shah, M. C. Crair, Retinocollicular synapse maturation and plasticity are regulated by correlated retinal waves. *J Neurosci* **28**, 292-303 (2008).

46. E. S. Ruthazer, J. Li, H. T. Cline, Stabilization of axon branch dynamics by synaptic maturation. *J Neurosci* **26**, 3594-3603 (2006).

47. A. Aggarwal *et al.*, Glutamate indicators with improved activation kinetics and localization for imaging synaptic transmission. *Nat Methods* **20**, 925-934 (2023).

48. M. P. Meyer, S. J. Smith, Evidence from in vivo imaging that synaptogenesis guides the growth and branching of axonal arbors by two distinct mechanisms. *J Neurosci* **26**, 3604-3614 (2006).

49. L. Luo. *Principles of Neurobiology* (Garland Science, ed. 2, 2020).

50. I. J. Kim, Y. Zhang, M. Meister, J. R. Sanes, Laminar restriction of retinal ganglion cell dendrites and axons: subtype-specific developmental patterns revealed with transgenic markers. *J Neurosci* **30**, 1452-1462 (2010).

51. Y. K. Hong, I. J. Kim, J. R. Sanes, Stereotyped axonal arbors of retinal ganglion cell subsets in the mouse superior colliculus. *J Comp Neurol* **519**, 1691-1711 (2011).

52. J. E. Vaughn, Fine structure of synaptogenesis in the vertebrate central nervous system. *Synapse* **3**, 255-285 (1989).

53. D. Barson *et al.*, Simultaneous mesoscopic and two-photon imaging of neuronal activity in cortical circuits. *Nat Methods* **17**, 107-113 (2020).

54. M. Pachitariu *et al.*, Suite2p: beyond 10,000 neurons with standard two-photon microscopy. *bioRxiv*, (2017).

55. P. Coupe, M. Munz, J. V. Manjon, E. S. Ruthazer, D. L. Collins, A CANDLE for a deeper in vivo insight. *Med Image Anal* **16**, 849-864 (2012).

56. C. Arshadi, U. Gunther, M. Eddison, K. I. S. Harrington, T. A. Ferreira, SNT: a unifying toolbox for quantification of neuronal anatomy. *Nat Methods* **18**, 374-377 (2021).
